# Anterograde Activin Signaling Regulates Postsynaptic Membrane Potential and GluRIIA/B Abundance at the *Drosophila* Neuromuscular Junction

**DOI:** 10.1371/journal.pone.0107443

**Published:** 2014-09-25

**Authors:** Myung-Jun Kim, Michael B. O’Connor

**Affiliations:** Department of Genetics, Cell Biology and Development, University of Minnesota, Minneapolis, Minnesota, United States of America; University of Sydney, Australia

## Abstract

Members of the TGF-β superfamily play numerous roles in nervous system development and function. In *Drosophila*, retrograde BMP signaling at the neuromuscular junction (NMJ) is required presynaptically for proper synapse growth and neurotransmitter release. In this study, we analyzed whether the Activin branch of the TGF-β superfamily also contributes to NMJ development and function. We find that elimination of the Activin/TGF-β type I receptor *babo,* or its downstream signal transducer *smox,* does not affect presynaptic NMJ growth or evoked excitatory junctional potentials (EJPs), but instead results in a number of postsynaptic defects including depolarized membrane potential, small size and frequency of miniature excitatory junction potentials (mEJPs), and decreased synaptic densities of the glutamate receptors GluRIIA and B. The majority of the defective *smox* synaptic phenotypes were rescued by muscle-specific expression of a *smox* transgene. Furthermore, a mutation in *actβ*, an Activin-like ligand that is strongly expressed in motor neurons, phenocopies *babo* and *smox* loss-of-function alleles. Our results demonstrate that anterograde Activin/TGF-β signaling at the *Drosophila* NMJ is crucial for achieving normal abundance and localization of several important postsynaptic signaling molecules and for regulating postsynaptic membrane physiology. Together with the well-established presynaptic role of the retrograde BMP signaling, our findings indicate that the two branches of the TGF-β superfamily are differentially deployed on each side of the *Drosophila* NMJ synapse to regulate distinct aspects of its development and function.

## Introduction

Activin/TGF-β and BMP-type ligands of the transforming growth factor-β (TGF-β) superfamily are well-known players in vertebrate neurogenesis [1]–[3] and neuroprotection [4]. Recent findings from both *in vitro* and *in vivo* studies also emphasize that the BMP and Activin/TGF-β pathways play important roles in the development of post-mitotic neurons, including neurite and dendritic outgrowth, modulation of synaptic physiology, and regulation of cognitive behavior (reviewed in [5].

Consistent with their roles in post-mitotic neuronal development and synaptic modulation, the ligands and signaling components of the BMP and Activin/TGF-β subfamilies are widely expressed in the mammalian adult brain and components of both subfamilies are often co-expressed in multiple sectors of the brain with particular enrichment found in the CA and DG hippocampal subregions [6]–[10]. Coexpression of BMP and Activin ligands and likely co-activation of both pathways in specific regions of the brain raises an intriguing question as to how these two pathways divide their roles in controlling neuronal development and function. Parsing out the relative contribution of each subfamily is difficult in the mammalian system, largely due to the extensive redundancy at multiple levels within the signaling pathways.

The TGF-β superfamily is evolutionally conserved and ligands of both the BMP and Activin/TGF-β sub-branches, as well as all canonical signal transduction components for each branch, are found in *Drosophila,* but with less redundancy than found in most vertebrate systems (reviewed in [11]). In *Drosophila*, retrograde BMP signaling is known to promote synaptic growth and to confer synaptic homeostasis at the larval NMJ [12]–[16]. The Activin/TGF-β pathway has also been implicated in regulating various aspects of *Drosophila* neuronal development and function including axon guidance and remodeling [17]–[20], as well as neuroblast proliferation in the larval brain [21]. Interestingly, muscles and motor neurons also express different Activin/TGF-β-type ligands [11], [18], but their roles in regulating NMJ development or function are less well characterized.

In this study, we have addressed the developmental and functional significance of the co-expression of Activin/TGF-β and BMP-type ligands in the *Drosophila* neuromuscular system using loss-of-function mutations in several of the Activin-like ligands as well as *babo* and *smox,* the Activin-specific type I receptor and R-Smad, respectively. Our results demonstrate that Activin/TGF-β signaling is essential for achieving normal synaptic densities of GluRIIA, GluRIIB and Dlg, whereas it is dispensable for synaptic growth at the NMJ. Consistent with the reduction of GluR densities at the NMJ, electrophysiological recordings reveal that *babo* and *smox* mutants exhibit smaller amplitudes and lower frequencies of mEJPs. Evoked synaptic transmission, however, was not altered in these mutations implying that mechanism(s) acting to maintain synaptic homeostasis are intact. In contrast, mutations in BMP pathway components either had no effect or increased synaptic GluR densities. Overall, our results, together with previous findings, suggest that the two TGF-β superfamily branches control multiple aspects of NMJ synaptogenesis from several different directions.

## Materials and Methods

### Fly strains and husbandry

Fly stocks were maintained on a standard cornmeal-yeast-agar medium at 25°C. For all experiments, the larvae were raised on apple juice-agar plates supplemented with yeast paste since the *babo* and *smox* larvae grow poorly on standard medium [22].

A *yw* stock was used as a wild-type control. The *babo^fd4^* allele produces a truncated receptor lacking the kinase domain [17]. *babo^df^* is a deficiency line that deletes the *babo* gene. The *smox^f4^* allele is a protein null allele [23]. The *daw^1^* and *daw^11^* mutations are null alleles [11], [18]. *actβ^ed80^* has been described previously and is presumed to be a null mutation [21]. *myo^1^* and *myo^4^* are deletion mutants generated by an FRT-mediated inter-chromosomal recombination technique [20]. The *wit^A12^* and *wit^B11^* alleles are described in [13]. The *mad^1^* and *mad^12^* were isolated in separate EMS screens and behave as genetic nulls [15], [24]. *med^3^* [25] and *med^c246^* [26] are loss-of-function alleles. The *actβ*-GAL4, *daw*-GAL4 and *myo*-GAL4 lines are described in [20], [21], [27]. Other stocks used include: UAS-*smox* [28], UAS-*babo^QD^* line 1A [29], Mhc-GluRIIA-myc [30], UAS-GluRIIA^GFP^ and UAS-GluRIIB^GFP^ [31], *Mef2*-GAL4 (Bloomington #27390), UAS-Cherry-Tub (Bloomington #2577) and *OK6*-GAL4 [12].

### Electrophysiology

Size-matched wandering stage larvae were washed in ddH_2_O and pinned down at the head and tail in a custom-made chamber filled with Ca^2+^-free HL3 saline. An incision was made along the dorsal midline and the body wall was pinned flat. All the visceral organs were then removed and the segmental nerves were cut near the ventral nerve cord. After rinsing, the bathing solution was replaced with 1 mM Ca^2+^ HL3 saline. Muscle 6 of abdominal segment 2 or 3 was impaled with a microelectrode having a resistance between 15 MΩ and 25 MΩ with 3 M KCl filling. For stimulation of motor neuron axons, the cut end of the segmental nerve was taken into a suction electrode. Depolarizing pulses of 1 ms duration were generated with a S88 Stimulator (Astro-Med, Inc), and were passed through the suction electrode at 0.1 Hz. Electrical signals were amplified and filtered at 1 kHz via Axoclamp 2B amplifier (Axon Instruments), digitized by Digidata 1322A (Axon Instruments), and recorded with Axocope 10 software (Axon Instruments). Only recordings with a resting membrane potential less then −60 mV were included in the analysis. Measurement of the mEJP and evoked events were performed using Mini Analysis (Synaptosoft Inc).

### Immunohistochemistry

Wandering stage larvae were picked and dissected as described above. The larval fillets were then fixed in Bouin’s fixative (Sigma) for 10 min with the dissecting plate on ice. For GluRIIA staining, the fixation was shortened to 2 min. The fixed fillets were incubated with primary antibody at 4°C overnight and with secondary antibody at RT for 2 hr. The following mouse monoclonal antibodies were obtained from the Developmental Studies Hybridoma Bank (DSHB) and used at the indicated dilutions: anti-GluRIIA (1∶50), anti-Dlg (1∶500), anti-Brp (1∶100). Other antibodies used include: mouse anti-Flag (1∶1000, Sigma), rabbit anti-GluRIIB and anti-GluRIIC (1∶2500, kind gifts from Aaron DiAntonio at Washington University School of Medicine, St. Louis, MO), rabbit anti-dPak (1∶500, a kind gift from S. Lawrence Zipursky at University of California, Los Angeles, CA), FITC-conjugated anti-HRP (1∶100, Jackson Immunoresearch Labs). Alexa488- and Alexa555-conjugated secondary antibodies (Molecular Probes) were used at 1∶200.

All images were acquired using a Zeiss LSM 710 confocal microscope. For assessing NMJ size, HRP stained boutons (both Ib and Is) on NMJ 6/7 of abdominal segments 2 and 3 were counted and normalized to the muscle surface area. The data are then expressed as boutons/10^4 ^µm^2^. For quantification of synaptic molecule abundance between control and mutant samples, fixation and antibody incubation were performed identically and the samples were imaged at the same exposure settings. Fluorescence intensities at the synaptic region were measured using Image J (NIH) software. The data are expressed as % of the wild-type.

### Immunoblot analysis

Ten 3^rd^ instar larvae per genotype were dissected in 1X PBS to remove internal organs and brain. The remaining muscle-epidermis complexes were homogenized in 21 µl of RIPA buffer (Sigma) supplemented with protease inhibitor (Complete mini; Roche). The samples were then incubated at 4°C for 40 min with agitation and centrifuged at 4°C. A 13 µl aliquot of supernatant was taken from each sample and incubated with 7 µl of 3X SDS loading buffer at 95°C for 5 min. An 8 µl aliquot out of 20 µl of final volume was loaded onto 4–12% Bis-Tris gels (Novex) and transferred to PVDF membranes (Millipore). The membranes were then incubated with mouse anti-GluRIIA (1∶50, DSHB) and mouse anti-α-Tubulin (1∶10000, Sigma) antibodies overnight at 4°C. HRP-conjugated anti-mouse IgG (1∶10000, Abcam) was used as a secondary antibody. Pierce ECL Western Blotting Substrate (Thermo Scientific) was used to visualize the bands. Band intensity was quantified using Image J (NIH) software.

### qRT-PCR

Larvae were dissected to remove internal organs and brain. Total RNA was extracted from the remaining muscle-epidermis complexes using TRIzol reagent (Invitrogen) and further cleaned via RNeasy Mini kit (Qiagen). First-strand cDNA was made from 1 µg total RNA using Superscript III first-strand synthesis kit (Invitrogen). qRT-PCR reactions were run on LightCycler 480 (Roche) in triplicate for each sample. *rp49* was used as an internal control. Cp values were obtained by 2^nd^ derivative maximum method. ΔCp values were calculated by subtracting Cp value of *rp49* from those of other genes. The fold changes were then obtained by 2^−ΔΔCp^ where ΔΔCp = ΔCp (gene of interest) − ΔCp (wild-type). Data are mean ± SEM from at least three separate mRNA extractions.

### Statistical Analyses

Comparisons between multiple groups were performed using One-way ANOVA followed by Dunnett’s multiple comparison test and results are designated with *’s. Unpaired t-tests were used to compare between two genotypes and resuts are designated with #’s. *P*-values less than 0.05 were considered to be significantly different. All statistical analyses were performed using Prism software (version 6.0, GraphPad Software).

## Results

### Activin/TGF-β signaling is not involved in regulating NMJ growth

**Figure 1:**
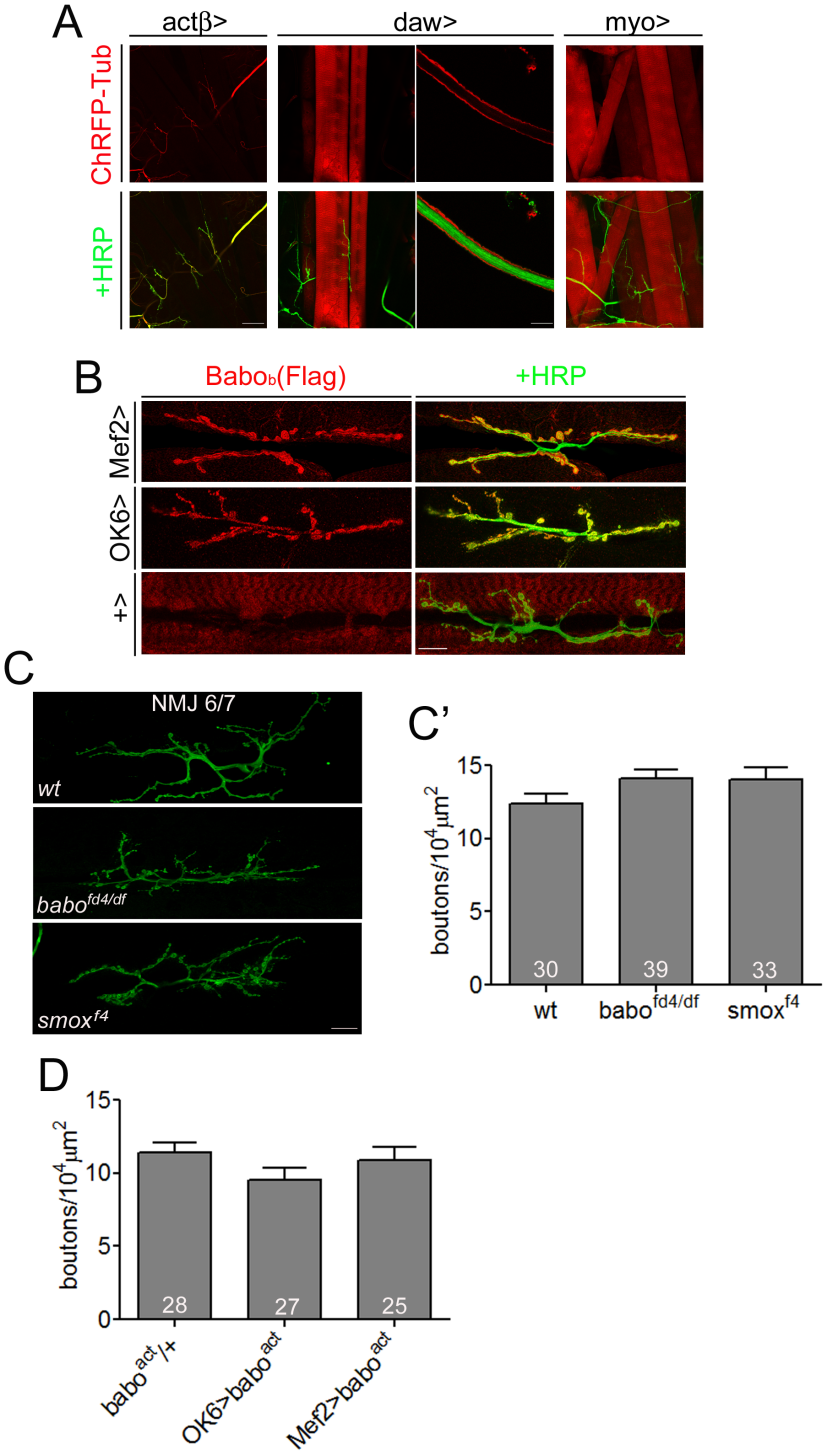
Activin/TGF-β pathway has no influence on synaptic growth at the NMJ. (A) Expression of Activin/TGF-β-type ligands in the neuromuscular system: *actβ-*GAL4-driven ChRFP-Tubulin expression is found in the axons and axon terminals innervating the body-wall muscles, *daw*-GAL4 drives Cherry-Tubulin expression at high levels in muscle 6/7 and surface nerve wrapping glial cells. Lower expression is found in muscle 12&13. *myo*-GAL4 drives Cherry-Tubulin expression in all body wall muscles. (B) Flag-tagged Babo_b_ was expressed in muscle by *Mef2*-GAL4 or in motor neurons by *OK6*-GAL4. Immunohistochemistry using anti-Flag antibody reveals preferred localization of Babo_b_(Flag) to NMJ both in the presynaptic and postsynaptic cells. (C) Representative images of the NMJ of *wt*, *babo^fd4/df^* and *smox^f4^* animals from HRP staining. In this figure, *babo^fd4/df^* and *smox^f4^* NMJs show concentrated distribution of synapses. (C’) Quantification of the NMJ size. The total number of synapses were counted and normalized to muscle surface area. There was no significant difference between the wild-type and *babo* or *smox* mutants. (D) Quantification of the NMJ size of the animals overexpressing constitutively-activated Babo either in the motor neuron or in the muscle. Overexpression of the activated Babo did not affect the NMJ size. Error bars represent SEM. Left scale bar in A is 80 µm; right scale bar in A is 16 µm; all others are 20 µm.

The Activin/TGF-β subfamily in *Drosophila* is composed of three ligands, Activin-β (Actβ), Dawdle (Daw) also know as Activin-like protein (Alp), and Myoglianin (Myo). Previous studies have demonstrated both pre- and postsynaptic expression of these ligands at the NMJ using *in situ* hybridization [17], [18], [29], [32]. To examine the NMJ expression pattern of the Activin/TGF-β-like ligands in more detail, we utilized enhancer/promoter GAL4 lines of *actβ*, *daw* and *myo* regulatory fusions to drive expression of UAS-Cherry-Tubulin. It has been shown previously that these GAL4 lines reproduce much of the endogenous expression patterns of these genes as revealed by *in situ* hybridization [18], [20], [21], [29]. In the third instar larva, *actβ*-GAL4-driven Cherry-Tubulin expression was detected in the axons and synapses of the motor neurons, but not in muscles (Fig. 1A), suggesting that at the NMJ *actβ* expression is limited to presynaptic motor neurons. Since TGF-β2 is shown to be transported along the neural processes and released via a Ca^2+^-dependent regulated pathway at termini distal to the cell body in PC12 cells and cultured hippocampal neurons [33], it is possible that Actβ may also be similarly transported and may play a role in regulating NMJ synaptic development or function. For *daw*, expression is seen in a subset of muscles with the highest intensity in muscles 6 and 7 of each hemisegment (Fig. 1A). Expression is also found in the surface glial cells that wrap the segmental nerves (Fig. 1A). As described previously [29], we found that *myo* expression is observed in all the body wall muscles (Fig. 1A) and also subsets of glia in the brain, but not the motor neuron wrapping glia.

Since different Activin/TGF-β ligands are expressed both pre- and postsynaptically at the NMJ, we were curious if Babo, the only type I receptor for Activin-like ligands, shows differential localization at the NMJ. Since there is presently no antibody available that detects endogenous Babo, we asked whether tissue-specific expression of Flag-tagged *babo_b,_* was able to localize to the NMJ. As illustrated in Figure 1B, we found that Flag-tagged Babo_b_ was enriched at the NMJ synapse regardless of whether a muscle- or motor neuron-specific GAL4 driver was used, suggesting that both pre- and postsynaptic mechanisms exist to enrich Babo localization at the NMJ, at least under over-expressed conditions.

We next asked if the Activin/TGF-β pathway promotes NMJ growth as found for the closely-related BMP pathway [12]–[14]. We examined the effect of mutations in both *babo* and *smox*, the two primary determinants of Activin pathway specificity. As shown in Fig. 1C, NMJs of *babo* and *smox* mutants tend to concentrate into a smaller area, however the normalized bouton number (both 1b and 1 s) is not significantly changed in these mutants compared to the wild-type control (Fig. 1C’). We also examined the effect of expressing a constitutively active form of Babo in either the muscle or the motor neuron and observed no alteration in NMJ size (Fig. 1D). Taken together, these results strongly argue that, in contrast to the BMP pathway, the Activin/TGF-β branch has little or no influence on synaptic growth at the NMJ. However, a previous report has argued that *babo* and *smox* mutants inhibit NMJ growth [34]. In that study, the authors used a different set of alleles (*babo^32/52^* transheterozygotes and *smox^388^*) and counted only the Ib boutons. We performed a similar assay using the *babo^32/52^* heteroallelic combination and counted only Ib boutons, as identified by high intensity of Dlg staining (Fig. S1A and B), and found that the *babo^32/52^* heteroallelic combination indeed exhibited a decreased number of Ib boutons in NMJ 6/7 (Fig. S1A′ and A″) and NMJ 4 (Fig. S1B′ and B″) as previously reported. However, the decrease in the numbers of Ib boutons was not observed when *babo^32^* was used over a genetically independent deficiency chromosome, *babo^df^* (Fig. S1). Since the *babo^32^* and *babo^52^* alleles share the same chromosome of origin (both were derived from excision of the same P element [29]), we suspect that the background chromosome from which *babo^32^* and *babo^52^* alleles were derived has other genetic variants that, when homozygosed, account for the Ib bouton number reduction. We also examined *babo^fd4/df^* and *smox^f4^* alleles using this alternative method, and again observed no significant change in the Ib bouton numbers (Fig. S1). In addition to the lack of change in the Ib numbers, p-Mad level in the motor neurons and *gbb* transcripts in the muscle-epidermis complexes were not reduced in *babo^fd4/df^* and *smox^f4^* mutants (Fig. S2), suggesting that Activin/TGF-β signaling is not regulating muscle-specific *gbb* expression as previously reported [34]. When considering both sets of data, our conclusion is that the Activin/TGF-β pathway does not play a significant role in regulating NMJ growth.

### Mutations in Activin/TGF-β pathway cause defective synaptic transmission

**Figure 2:**
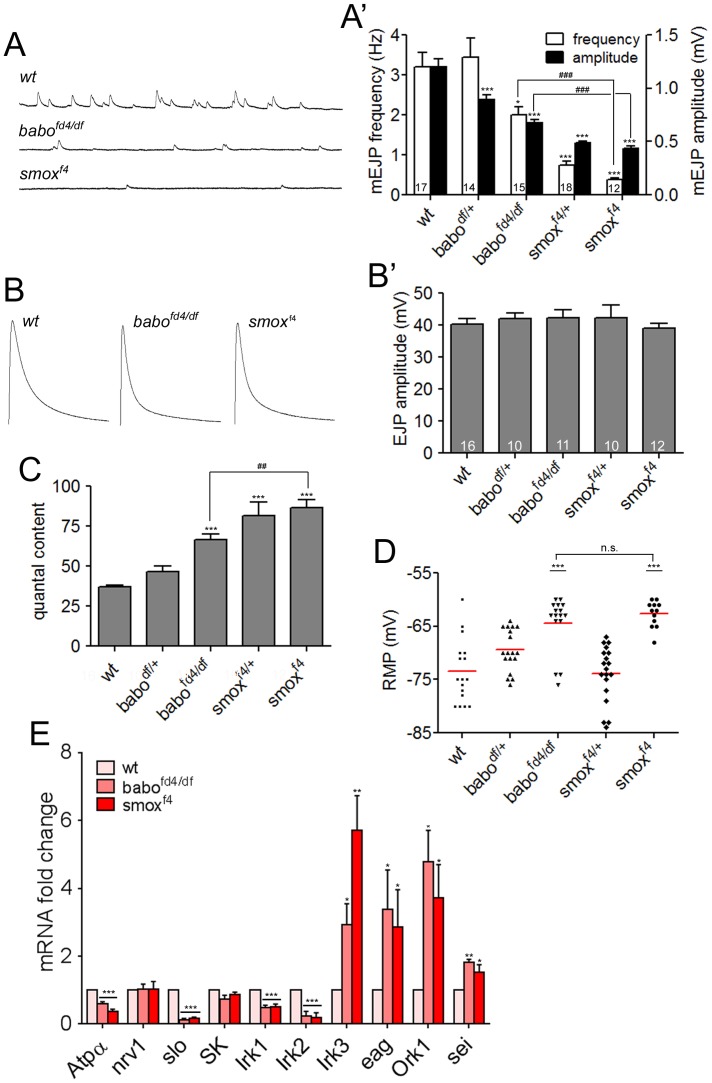
Activin/TGF-β pathway mutants exhibit defective synaptic activity. (A and A′) Representative traces and quantification of mEJPs of wild-type and Activin/TGF-β pathway mutants. Note that both the size and frequency of the mEJPs are decreased in *babo* and *smox* mutants. (B and B′) Representative traces and quantification of EJP in Activin/TGF-β pathway mutants. The mean peak amplitude is not affected by the *babo* and *smox* mutations. (C) Quantification of the quantal content. Note the increased quantal content in *babo^fd4/df^* and *smox^f4^* animals. (D) Distribution of resting membrane potentials. The *babo* and *smox* mutants exhibit more depolarized resting membrane potentials. (E) Relative transcript levels of the molecules potentially involved in regulating the resting membrane potential revealed by qRT-PCR assay. Error bars represent SEM. *p<0.05, **p<0.01, ***p<0.001 from One-way ANOVA followed by Dunnett’s multiple comparison test. ##p<0.01, ###p<0.001 from unpaired t-test. Each genotype was compared to wild-type. Additionally, *smox^f4^* was also compared to *babo^fd4/df^* in A′, C and D as indicated by lines.

**Figure 3:**
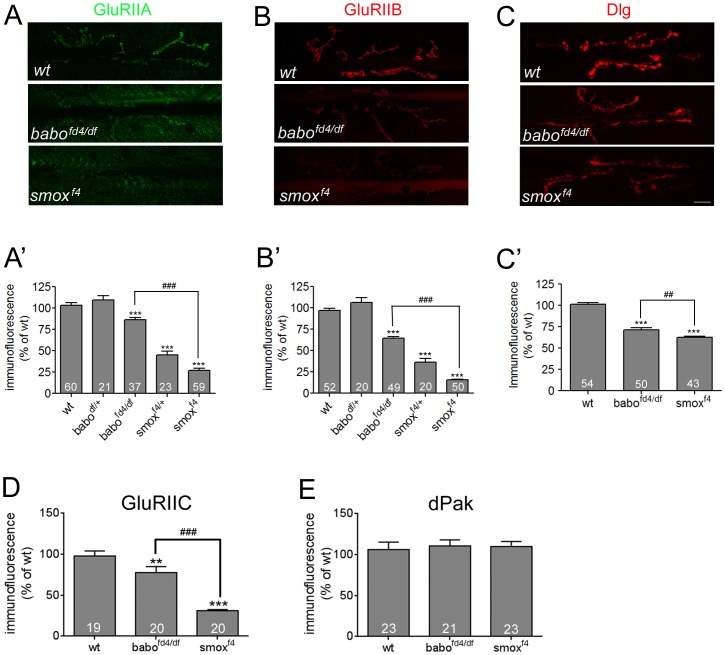
Postsynaptic densities of GluRIIA, GluRIIB and Dlg are decreased in Activin/TGF-β pathway mutants. (A and A’) Representative confocal images of the GluRIIA staining and quantification. The GluRIIA abundance is reduced greatly in *smox^f4^* and slightly in *babo^fd4/df^*NMJs. (B and B′) Representative confocal images of the GluRIIB staining and quantification shows decreased abundances in *babo^fd4/df^* and *smox^f4^* NMJs. (C and C′) Representative confocal images of Dlg staining and quantification. The Dlg level is also decreased in the *babo* and *smox* mutants. (D) Quantification of synaptic GluRIIC abundance in wild-type and *babo* and *smox* mutants. (E) Quantification of dPak staining reveals no difference between wild-type and Activin/TGF-β pathway mutants. Error bars represent SEM. *p<0.05, **p<0.01, ***p<0.001 from One-way ANOVA followed by Dunnett’s multiple comparison test. ##p<0.01, ###p<0.001 from unpaired t-test. Each genotype was compared to wild-type. Additionally, *smox^f4^* was also compared to *babo^fd4/df^* in A′, B′, C′ and D as indicated by lines. Scale bar equals 20 µm.

The observation that mutations in Activin/TGF-β signaling molecules do not impair synaptic growth still leaves open the possibility that this pathway might regulate more subtle aspects of the synapse function, which cannot be detected by the bouton-counting assays. Therefore, we performed electrophysiological recordings from wild-type and mutant muscles to look for alterations in various synaptic parameters. We found that the mEJP amplitude, which reflects the muscle’s response to neurotransmitter release from a single synaptic vesicle, was greatly decreased in *babo* and *smox* mutants with the *smox^f4^* animals exhibiting the most severe decrease (wt = 1.20±0.08 mV, *babo^fd4/df^* = 0.68±0.03 mV, *smox^f4^* = 0.43±0.02 mV; Fig. 2A and A’). In contrast to mEJP, the EJP was not affected by the mutations and showed similar mean amplitudes as wild-type (wt = 40.31±1.73 mV, *babo^fd4/df^* = 42.36±2.34 mV, *smox^f4^* = 39.00±1.50 mV; Fig. 2B and B’). Consequently, the quantal content obtained by dividing the amplitude of EJP with that of mEJP was increased in the *babo* and *smox* mutants (wt = 37.01±0.92 mV, *babo^fd4/df^* = 66.63±3.44 mV, *smox^f4^* = 86.67±4.74 mV; Fig. 2C). The unaltered EJP size indicates that retrograde homeostatic modulation of presynaptic release is not impaired in *babo* and *smox* mutants. We also plotted quantal content versus quantal size and found an inverse relationship between them (Fig. S3A). A similar result has been obtained in *glurIIA* mutants and PhTox-treated animals [35]. The inverse relationship indicates that quantal content scales to offset the changes in quantal size to maintain a constant level of EJP. In addition to the amplitude, the frequency of mEJP release was also decreased (wt = 3.20±0.36 Hz, *babo^fd4/df^* = 2.01±1.20 Hz, *smox^f4^* = 0.39±0.03 Hz; Fig. 2A and A’). Since there is no change in overall size of the NMJ in *babo* and *smox* mutants, the reduced frequency indicates that the probability of spontaneous vesicle fusion per bouton is reduced assuming a constant number of fusion sites. However, it should also be noted that there is a technical possibility of losing mEJPs in the noise when the amplitude is extremely small leading to the lower frequency shown by *smox* mutants. Interestingly, the EJPs of *babo* and *smox* mutants showed more rapid decay kinetics compared to wild-type (decay time constant: wt = 22.7±0.56 ms, *babo^fd4/df^* = 13.9±0.23 ms, *smox^f4^ = *13.0±0.48 ms; Fig. 2B) in spite of the unaltered amplitude. We believe that the altered decay kinetics likely result from deregulated expression of potassium channels (Fig. 2E) in these mutants. A similar decrease in the decay time constant has been reported when Kir2.1, a human inward-rectifier potassium channel, is over-expressed in muscle [36]. In addition, the decrease in GluRIIA abundance (Fig. 3A and A′) may also contribute since a similar phenotype in the decay of EJP has been reported in *glurIIA* mutants [37].

While carrying out these recordings, we realized that larval muscles of *babo* and *smox* mutants tend to be depolarized compared to those of wild-type. The scattered-dot plot of resting membrane potentials clearly shows a depolarization in *babo* and *smox* muscles compared to wild-type (average resting membrane potential: wt = −73.47±1.49 mV, *babo^fd4/df^* = −64.44±1.31 mV, *smox^f4^* = −62.67±0.70 mV; Fig. 2D). To address the underlying mechanism, we performed a qRT-PCR analysis to determine if there is a change in the mRNA levels of several molecules implicated in membrane physiology. We chose ten genes for this study and the name and molecular feature of the products are listed in Table S1. As shown in Fig. 2E, the transcript levels of *Atpα*, *slo*, *irk1* and *irk2* are significantly decreased in *babo* and *smox* mutants (Fig. 2E). In contrast, the transcripts of *irk3*, *eag*, *ork1* and *sei* are greatly up-regulated whereas *nrv1* and *SK* transcript levels are unaltered in the *babo* and *smox* mutants (Fig. 2E). It is not understood at present if and how this disregulation leads to the depolarized resting membrane potential. Nevertheless, the data indicate that Activin/TGF-β signaling plays a crucial role in the regulation of potassium channel as well as Na^+^/K^+^-ATPase expression levels and thereby profoundly affects the muscle resting membrane potential.

### Activin/TGF-β signaling controls the postsynaptic densities of GluRIIA, GluRIIB and Dlg

Previous work has established that the amplitude of mEJP in *Drosophila* larval muscles is largely determined by the abundance of GluRs available at the postsynaptic region [30], [37]. There are two types of GluRs in *Drosophila* larval muscles, A-type containing GluRIIA subunit and B-type containing GluRIIB subunit [31]. The abundance of each type of GluR was investigated by immunostaining using available antibodies raised against GluRIIA and GluRIIB [38]. In addition, the level of Dlg, a scaffold protein that recruits diverse molecules to the postsynaptic region, was also examined.

We found that the density of GluRIIA at the third instar larval NMJ, as quantified by fluorescent intensity, is strongly reduced in *smox* null mutants (27.15±2.09% of wt, Fig. 3A and A’). We also observed a significant decrease in GluRIIA levels in the *smox^f4/+^* heterozygous animals (45.37±4.0% of wt). A statistically significant decrease of GluRIIA levels was also seen in *babo* loss-of-function animals (81.23±2.46% of wt, Fig. 3A and A’), although it is not as severe, and *babo^df/+^* heterozygous NMJs showed no decrease.

We next examined the level of GluRIIB. Like GluRIIA, the abundance of GluRIIB subunit is greatly decreased at the NMJs of *smox* null and *smox^f4/+^* heterozygous animals (*smox^f4^* = 15.52±0.70% of wt, *smox^f4/+^* = 36.53±4.04% of wt; Fig. 3B and B’). The *babo* null animals exhibited a less severe, but statistically significant decrease (64.46±2.07% of wt, Fig. 3B and B’). Since both GluRIIA and GluRIIB subunits are decreased, we speculated that a reduced number of functional iGluRs exist at the NMJs of Activin/TGF-β pathway mutants [38], [39]. To test this idea, we examined the synaptic abundance of GluRIIC, an essential subunit of iGluR [38]. We observed that synaptic GluRIIC level is diminished greatly in *smox* and mildly in *babo* mutants (Fig. 3D). These results support the idea of diminished iGluR at the NMJs and correlate well with the difference in the severity of reduction in the GluRIIA and GluRIIB levels shown by *babo* and *smox* mutants. To investigate if the reduction in GluR density caused smaller mEJP sizes of *babo* and *smox* mutants (Fig. 2A and A′), we calculated quantal variances (CVs) from each genotype. CVs greater than 0.4 indicate that postsynaptic receptors are unsaturated by a quantal release due to the existence of a large number of receptors whereas CVs less than 0.2 indicate saturation [40], . The mean CV was significantly reduced in *babo* mutants (0.33±0.024 of *babo* vs. 0.58±0.02 of wt; Fig. S3B). Furthermore, the *smox* mutants showed a greater decrease in mean CV (0.21±0.47). Therefore, it appears that the postsynaptic receptors are likely saturated by a quantal release in *smox* and, to a less extent, in *babo* mutants. The result is consistent with the idea of diminished iGluR in *babo* and *smox* mutants and implies that the reduction in postsynaptic GluR density caused the smaller mEJPs.

**Figure 4:**
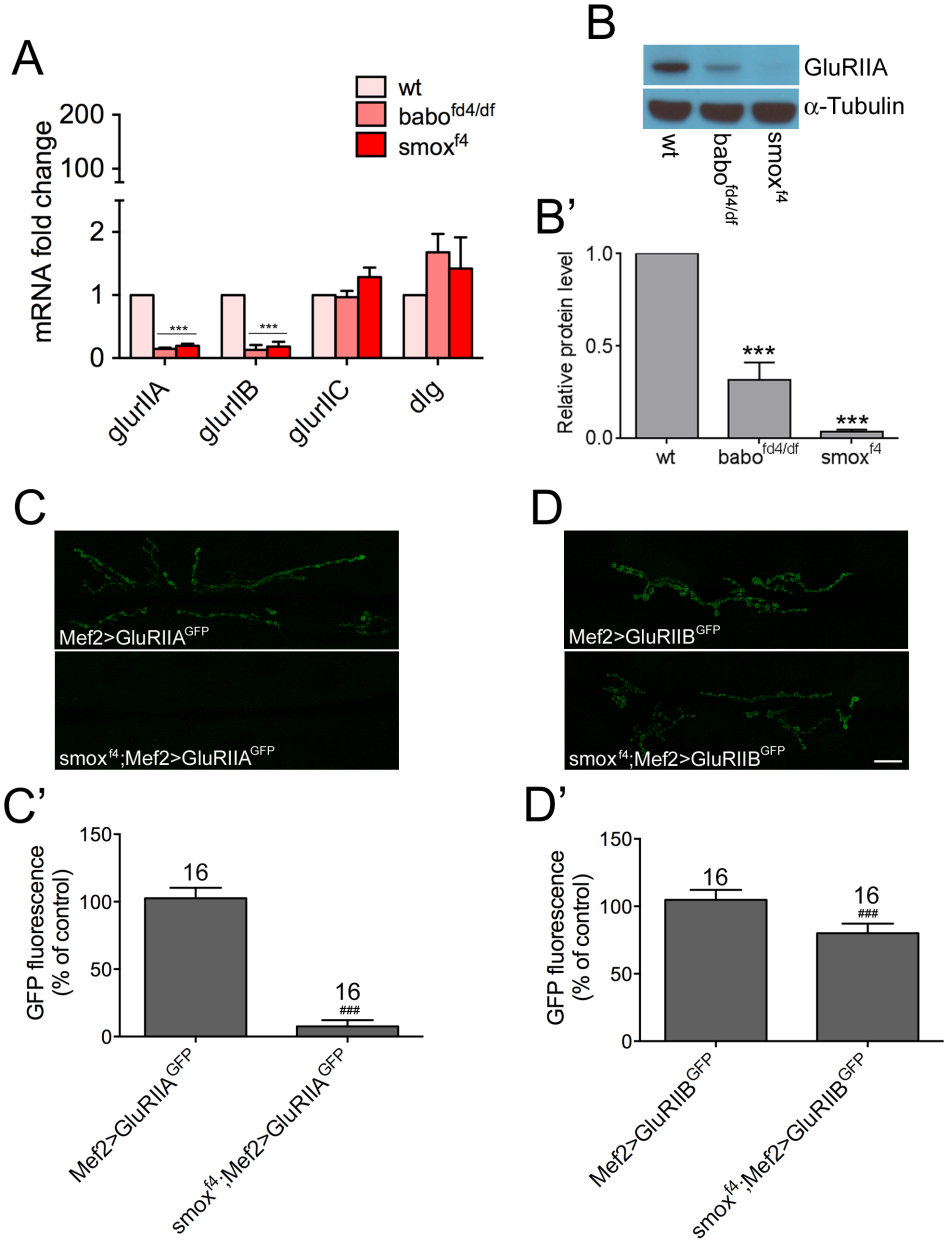
Synaptic GluRIIA and GluRIIB abundances are differentially regulated by Activin/TGF-β pathway. (A) Transcript levels of *glurIIA*, *glurIIB, glurIIC* and *dlg*. Results from qRT-PCR assay demonstrate significant decreases in the levels of *glurIIA* and *glurIIB* transcripts in *babo* and *smox* mutants. (B and B′) Total protein level of GluRIIA. A representative blot (B) and quantification (B′). (C and C′) Exogenous expression of *glurIIA-gfp* in wild-type and *smox* mutant backgrounds. Representative confocal images (C) and quantification (C′) of GFP activity at the synapses. (D and D′) Exogenous expression of *glurIIB-gfp*. Representative confocal images (D) and quantification (D′) of GFP activity at the synapses. ***p<0.001 from One-way ANOVA followed by Dunnett’s multiple comparison test. ###p<0.001 from unpaired t-test. Error bars represent SEM. Scale bar equals 20 µm.

Dlg staining at the NMJ of *babo* and *smox* mutant animals also exhibits a significant decrease (Fig. 3C and C’). In contrast to GluRIIA and GluRIIB, the Dlg level was similarly decreased in *babo* and *smox* mutant animals (*babo^fd4/df^* = 71.14±2.47% of wt, *smox^f4^* = 62.32±1.64% of wt). Despite the significant reduction in the density of Dlg at the NMJ, mRNA levels were not altered in the muscle-epidermis preparations (Fig. 4A). Consistent with the unaltered mRNA level, Western blot analysis revealed that the total amount of the Dlg protein was comparable to that of wild-type in the muscle-epidermis preparations of *babo* and *smox* mutants (data not shown). Given that synaptic abundance is decreased without a change in the total amount of Dlg protein, we envision a defect in the mechanism regulating the transport of Dlg protein to the synapses. It has been shown that the synaptic targeting of Dlg is regulated by phosphorylation at the S48 residue by CaMKII [42] or the S797 site by PAR-1 kinase [43], and the result of phosphorylation at either site is delocalization of Dlg from the synapses. Based on these reports, we hypothesized that hyperactivity of CaMKII or PAR-1 kinase-dependent phosphorylation might underlie the reduced synaptic densities of Dlg shown in *babo* and *smox* mutant animals. To test this idea, we expressed RNAi transgenes of *camkII* and *par-1* to knockdown the protein expression and assessed the densities of Dlg at the synapses. The expression of RNAi transgenes, however, did not result in an increase in synaptic Dlg levels (data not shown). In an accompanying experiment, we expressed Ala, a pseudo-substrate of CaMKII, to inhibit the activity of CaMKII in *smox* null animals, but this treatment also failed to increase the synaptic Dlg level. Finally, we detected no change in PAR-1 protein level in *babo* and *smox* mutants by Western blot analysis (data not shown). It has been shown that a cascade of molecules involving *Drosophila* p21-activated kinase (dPak) act as postsynaptic signaling/scaffold proteins in parallel with Dlg-mediated protein localization [44]. We examined if the dPak abundance at the NMJ of wild-type and Activin/TGF-β pathway mutants is altered similar to Dlg. As shown in Fig. 3E, we observed no change in the synaptic level of dPak. Collectively, these results led us to conclude that the Activin/TGF-β pathway does not have a general effect on the localization of postsynaptic scaffold proteins but instead specifically regulates synaptic targeting of certain components, such as Dlg, via an unknown mechanism that does not involve CaMKII or PAR-1 kinase.

We also examined the abundance of Bruchpilot (Brp), an active zone-organizing protein [45], in an effort to determine if Activin/TGF-β signaling influences the accumulation of presynaptic molecules. In contrast to the postsynaptic components, deposition of Brp at the synapses was not decreased in the *babo* and *smox* mutants (Fig. S4A and A′). Additionally, we counted the Brp puncta that have been proposed to represent active zones. The number of Brp puncta was then normalized to the synaptic area delineated by HRP staining. We focused on muscle 4 since Ib and Is boutons are usually separated in these muscles. As shown in Fig. S4B insets, enlargement of the Brp-staining pictures at the bouton area reveals discrete and round-shaped puncta both in wild-type and Activin/TGF-β pathway mutants consitent with formation of normally shape active zones. Furthermore, the number of Brp puncta per synaptic area was not changed in *babo* and *smox* animals (Fig. S4B and B′) implicating normal density of active zones in these mutants. Taken together, these results support the idea that Activin/TGF-β signaling is primarily involved in regulating postsynaptic development and not presynaptic release site formation.

### Activin/TGF-β pathway regulates synaptic GluRIIA and GluRIIB abundances through different mechanisms

Considering that Smox is a transcriptional regulator, an obvious possibility is that an alteration in the transcript level of *glurIIA* and *glurIIB* underlies the decrease in synaptic density. Quantative RT-PCR analysis was performed to assess the transcript levels of *glurIIA* and *glurIIB*. We observe a great decrease in the amount of *glurIIA* transcript both in *babo* and *smox* muscles (Fig. 4A). In accordance, the total protein level of GluRIIA is also found to be diminished (Fig. 4B and B′). We also observe that the *glurIIB* transcirpt is decreased in *babo* and *smox* mutants (Fig. 4A). In contrast, the transcript level of *glurIIC* is not changed (Fig. 4A), suggesting that the decrease in the synaptic density of GluRIIC (Fig. 3D) is likely due to the lower availability of GluRIIA and GluRIIB subunits whose transcirpts are greatly decreased in Activin/TGF-β pathway mutants. Finally, the transcript level of *dlg* is not changed (Fig. 4A) which is consistent with the unaltered total protein level (data not shown).

The concomitant decrease in the transcript and total protein levels (Fig. 4A and B) indicates that the synaptic GluRIIA abundance is mainly determined by the transcript level in the Activin/TGF-β pathway mutants. To test if the transcript level of *glurIIA* is determined by transcriptional regulation or if there is a post-transcriptional mechanism involved, we exogenously expressed *gfp*-tagged *glurIIA* transgene using UAS-GAL4 system in the *smox* mutant as well as in wild-type backgrounds and examined synaptic GFP activity. We reasoned if the mRNA level is solely determined by transcriptional regulation, expression of *glurIIA* using an exogenous promoter and transcriptional activator should achieve the same transcript and protein levels in wild-type and Activin/TGF-β pathway mutants. Interestingly, we observe almost no GFP signal at the synapses of *smox* mutant animals (Fig. 4C and C′). A similar result was obtained in an independent experiment using a different transgene (Fig. S5). Therefore, it appears that the expression of *glurIIA* is actively suppressed in Activin/TGF-β pathway mutants not only from the endogenous gene but also from an exogenous transgene. In a parallel experiment, we expressed *gfp*-tagged *glurIIB* in the same way as *glurIIA-gfp* expression. Unlike for the *glurIIA-gfp* transgene, a significant increase in GFP flouresence is found at the synapses of *smox* mutants (Fig. 4D and D′) when the *glurIIB-gfp* transgene is overexpressed in muscles. The GFP signal reached 82.62±2.31% of that shown by control animals, which is a great increase from the level of endogenous GluRIIB (15.52±0.70% of wt; Fig. 3B and B′) in *smox* mutant larvae. These results imply that the abundance of synaptic GluRIIB subunits in *smox* and *babo* mutants is primarily due to a transcriptional defect. Collectively, these results suggest that the Activin/TGF-β pathway regulates synaptic abundance of GluRIIA and GluRIIB through different mechanisms, consistent with previous studies [46], [47].

### Muscle-specific expression of *smox* rescues the defects in protein localization and synaptic activity

**Figure 5:**
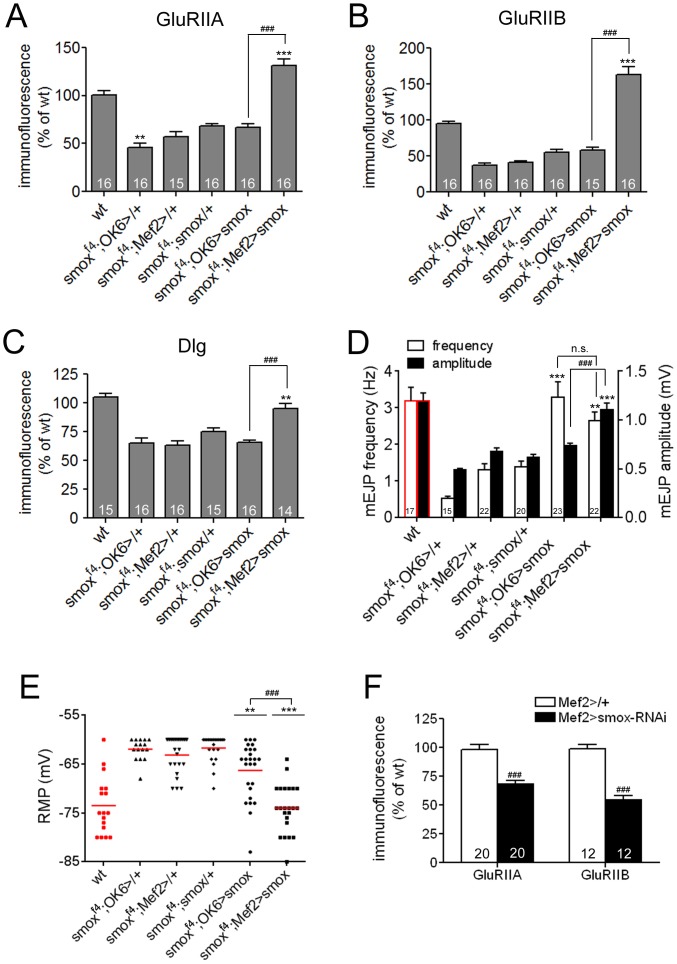
Muscle expression of *smox* transgene rescues the defective phenotypes of *smox* mutants. (A) Quantification of GluRIIA abundance. The decreased GluRIIA level exhibited by s*mox* mutants is rescued over the level of control animals (*smox^f4^;*UAS-*smox/+*) and even the wild-type only when the *smox* transgene is expressed via the muscle driver, *Mef2*-GAL4. (B) Quantification of GluRIIB level demonstrates a specific rescue by muscular expression of *smox* transgene. (C) The decreased level of Dlg is also restored by muscular expression of *smox* transgene. (D) Quantification of the size and frequency of mEJPs. Decreased mEJP size and frequency are restored in *smox^f4^;Mef2-*GAL4/UAS*-smox* animals. Note that expression of the *smox* transgene in motor neurons (*smox^f4^;OK6*-GAL4/UAS-*smox*) most effectively rescues the decreased mEJP frequency. (E) Depolarized resting membrane potential of the *smox* mutants is fully restored by muscular expression of *smox* transgene. (F) Quantification of GluRIIA and GluRIIB abundance in control (Mef2>/+) and *smox* knockdown (Mef2>*smox*-RNAi) animals. Error bars represent SEM. **p<0.01, ***p<0.001 from One-way ANOVA followed by Dunnett’s multiple comparison test. ###p<0.001 from unpaired t-test. Each genotype was compared to *smox^f4^;*UAS*-smox/+*. Additionally, *smox^f4^;Mef2*-GAL4/UAS-*smox* was also compared to *smox^f4^;OK6-*GAL4*/*UAS-*smox.* Data sets of wild-type shown in Fig. 2 were included in D and E for reference and marked with red.

Previous studies have implicated both pre- and postsynaptic mechanisms in regulating the localization of synaptic molecules as well as synaptic activities [48]. To determine the relative importance of pre- and postsynaptic Activin/TGF-β signaling in regulating the synaptic densities of GluRs and Dlg and various aspects of synaptic activity, we sought to restore Activin/TGF-β signaling to one side of the synapse in *smox* null animals by tissue specific expression of a UAS-*smox* transgene in muscle or the motor neuron using the *Mef2*-GAL4 and *OK6*-GAL4 driver lines, respectively. Control animals carried only the *smox* transgene or one of the two GAL4 drivers in the *smox* null background. While the presence of the UAS-*smox* transgene alone shows some rescue, presumably because of leaky expression, muscle specific induction using *Mef2*-GAL4 caused a large increase in the localization of synaptic molecules leading to a complete rescue of the phenotypes. For instance, the synaptic level of GluRIIA in *smox^f4^*; *Mef2*-GAL4/UAS-*smox* animals was 131.1±7.1% of wt (Fig. 5A). Likewise, GluRIIB level in *smox^f4^*; *Mef2*-GAL4/UAS-*smox* animals (162.8±11.1% of wt) was higher than those of wild-type and *smox^f4^*;UAS-*smox*/+ controls (55.3±3.9% of wt) by 62.8% and 107.5%, respectively (Fig. 5B). Finally, the level of Dlg in *smox^f4^*; *Mef2*-GAL4/UAS-*smox* animals (94.9±4.4% of wt) was comparable to that of wild-type and was higher than that of *smox^f4^*; UAS-*smox*/+ control larvae (75.3±3.0% of wt) by 19.3% (Fig. 5C). In contrast to the *Mef2*-GAL4-driven expression, *OK6*-GAL4-driven *smox* expression failed to increase the synaptic densities of GluRIIA, GluRIIB and Dlg over the levels shown in *smox^f4^*;UAS-*smox*/+ control animals (Fig. 5A, B and C). As an alternative means of testing the importance of postsynaptic Activin/TGF-β signaling, we expressed *smox-*RNAi transgene in the muscle to deplete Smox and observed a great reduction in the densities of GluRIIA and GluRIIB in the *smox-*RNAi muscle (Fig. 5F). Collectively, these results strongly suggest that postsynaptic and not presynaptic Activin/TGF-β signaling is essential to achieve normal postsynaptic densities of GluRIIA, GluRIIB and Dlg.

Given that the postsynaptic but not presynaptic expression of *smox* restores the levels of synaptic GluRs and Dlg, we asked if muscle-specific *smox* expression caused a concomitant change in the muscle response to synaptic activity. To answer this question, we recorded mEJPs from the animals in which Activin/TGF-β signaling was specifically restored to one side of the synapse and assessed the average amplitude of the mEJPs. As found for GluR and Dlg densities, the UAS-*smox* transgene itself rendered a slight increase in the average amplitude of mEJP compared to the level of *smox^f4^*; *OK6*-GAL4/+ larvae (0.62±0.03 mV of *smox^f4^*;UAS-*smox*/+ vs. 0.49±0.02 mV of *smox^f4^*;*OK6*-GAL4/+; Fig. 5D). Interestingly, the *Mef2*-GAL4 driver itself also caused an increase in the average amplitude (0.68±0.03 mV of *smox^f4^*;*Mef2*-GAL4/+ vs. 0.49±0.02 mV of *smox^f4^*;*OK6*-GAL4/+ animals; Fig. 5D). However, the effect of the UAS-*smox* transgene in combination with the *Mef2*-GAL4 driver showed a substantial additional increase in the average amplitude of mEJP in the *smox^f4^*;*Mef2*-GAL4/UAS-*smox* animals (1.10±0.07 mV) compared to *smox^f4^*; UAS-*smox*/+ or *smox^f4^*; *Mef2*-GAL4/+ controls (Fig. 5D). In contrast to muscle expression, motor neuron-specific expression of *smox* did not lead to any further increase in mEJP amplitude over the level seen in *smox^f4^*;UAS-*smox*/+ larvae (Fig. 5D). These results further confirm the importance of postsynaptic Activin/TGF-β signaling in controlling mEJP amplitude through regulation of postsynaptic GluRs levels.

In addition to amplitude, we also analyzed the frequency of mEJPs in various rescue contexts. Intriguingly, we observed a significant increase in the frequency of mEJPs when Activin/TGF-β signaling is restored in either the motor neurons or the muscle (3.28±0.42 Hz of *smox^f4^*;*OK6*-GAL4/UAS-*smox* vs. 1.39±0.15Hz of *smox^f4^*;UAS-*smox*/+ animals; and 2.65±0.23 Hz of *smox^f4^*;*Mef2*-GAL4/UAS-*smox* vs. 1.39±0.15 Hz of *smox^f4^*;UAS-*smox*/+ animals; Fig. 5D). This result suggests that both pre- and postsynaptic Activin/TGF-β signaling may control spontaneous synaptic vesicle fusion.

Lastly, we found that the depolarized resting membrane potential of the *smox* muscles was rescued to the wild-type level by post- but not presynaptic restoration of Activin/TGF-β signaling (−66.27±1.22 mV of *smox^f4^*; *OK6*-GAL4/UAS-*smox* and −74.00±1.03 mV of *smox^f4^*; *Mef2*-GAL4/UAS-*smox* animals vs. −73.47±1.4 mV of wt; Fig. 5E). In conclusion, our tissue specific rescue experiments demonstrate that postsynaptic Activin/TGF-β signaling plays a major role in controlling the synaptic densities of GluRs and Dlg, mEJP amplitude and resting muscle membrane potential; while either presynaptic or postsynaptic Activin/TGF-β signaling can affect spontaneous vesicle fusion frequency.

### A mutation in the Activin/TGF-β ligand *actβ* produces significant defects in the abundance of postsynaptic molecules and synaptic activity

**Figure 6:**
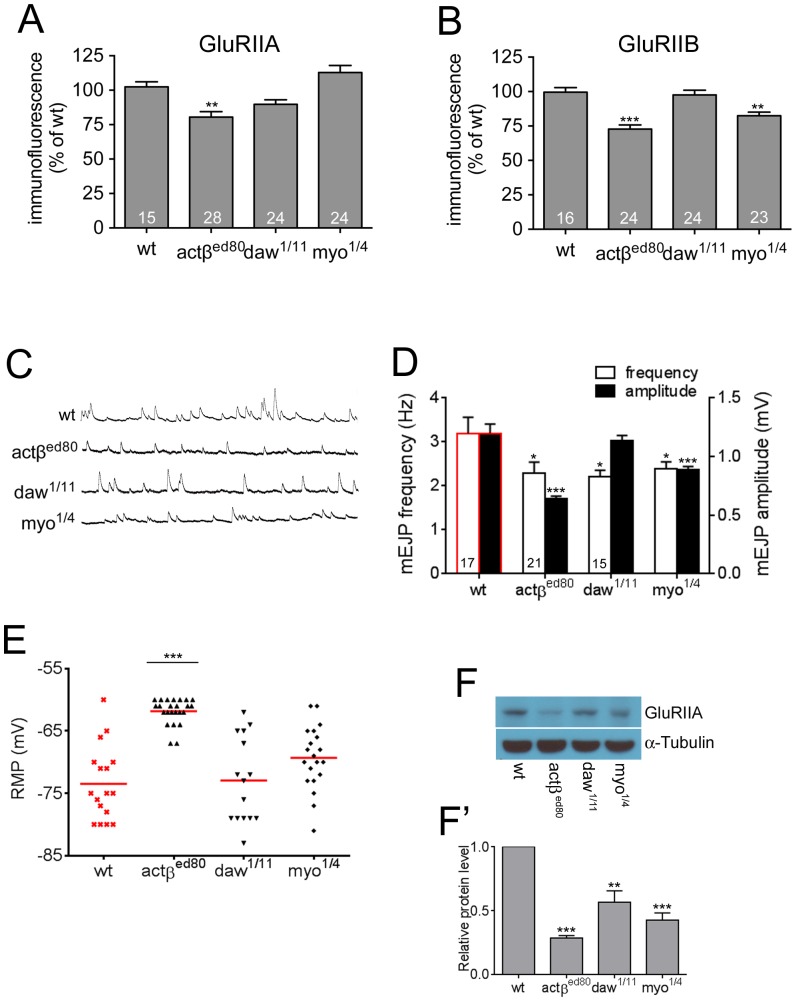
Among the Activin-type ligands, a mutation in *actβ* causes the most severe phenotypes. (A) Synaptic GluRIIA levels are decreased greatly in *actβ* and slightly in *daw* mutants but not in *myo* mutants. (B) Decreased GluRIIB levels in Activin-type ligand mutants. The greatest decrease is shown by *actβ* mutants. (C) Representative traces of mEJPs of wild-type and Activin/TGF-β ligand mutants. (D) Quantification of mEJP size and frequency. (E) Distribution of the resting membrane potentials. The *actβ* mutants show the most depolarized resting membrane potentials. (F and F′) Total protein level of GluRIIA. A representative blot image (F) and quantification (F′). Error bars represent SEM. *p<0.05, **p<0.01, ***p<0.001 from One-way ANOVA followed by Dunnett’s multiple comparison test. Each genotype was compared to the wild-type. Data sets of wild-type shown in Fig. 2 were included in D and E for reference and marked with red.

Given that all Activin-like ligands are expressed in either muscles, motor neurons, or motor neuron-wrapping glia [17], [18], [29], [32], [49], we wished to determine the relative contributions of each ligand to regulation of NMJ synaptic activity. Since *mav* has been examined previously [49], we focused our attention on the analysis of loss-of-function mutants in *actβ*, *daw* and *myo.* We first investigated the synaptic density of GluRIIA in these mutants. As shown in Fig. 6A, the *actβ^ed80^* homozygous mutants exhibit the greatest effect on the level of GluRIIA, decreasing it to 80.42±4.0% of wild-type which is comparable to that shown by *babo* null animals (81.23±2.46% of wt, Fig. 3A′). The *daw^1/11^* mutation also caused a reduction in the level of GluRIIA, but the decrease was smaller than seen in *actβ^ed80^* mutant background (89.78±2.74% of wt, Fig. 6A). Interestingly, *myo* null mutant animals (*myo^1/4^*) exhibited an elevated level of GluRIIA (112.9±5.04% of wt; Fig. 6A) even though *myo* is heavily expressed in muscles (Fig. 1A). An immunoblot analysis was also performed to investigate the total protein level of GluRIIA. The result shows that *actβ^ed80^* mutation causes the most significant decrease in total amount of GluRIIA (Fig. 6F and F′).

We next examined synaptic GluRIIB levels. As found for GluRIIA, *actβ^ed80^* animals displayed the greatest reduction in the synaptic GluRIIB level (72.71±3.04% of wt, Fig. 6B) while loss of *daw* or *myo* caused no or a less severe decrease in GluRIIB levels (*daw^1/11^* = 97.53±2.94% of wt, *myo^1/4^* = 82.41±2.64% of wt, Fig. 6B).

We also performed an electrophysiological analysis of synaptic activity in the Activin/TGF-β ligand mutants. As found for the densities of postsynaptic molecules, the *actβ^ed80^* mutation caused the most significant decrease in the average amplitude of the mEJP (0.64±0.01 mV, Fig. 6C and D) which is comparable to that shown by *babo* null animals (0.68±0.03 mV of *babo^fd4/df^*, Fig. 2A′). The *myo^1/4^* mutant combination slightly lowered the average amplitude of mEJP (0.88±0.03 mV, Fig. 6C and D) while the *daw^1/11^* mutation had a minimal effect (1.14±0.04 mV of *daw^1/11^* vs 1.20±0.08 mV of wt ). In addition, we also analyzed the frequency of mEJP. Unlike the amplitude, the average frequency of mEJP was decreased similarly among Activin/TGF-β ligand mutants (2.28±0.26 Hz of *actβ^ed80^*, 2.206±0.14 Hz of *daw^1/11^* and 2.38±0.16 Hz of *myo^1/4^* vs. 3.19±0.36 Hz of wt; Fig. 6C and D).

Lastly, we measured the resting membrane potential and found that *actβ^ed80^* mutants showed a significantly depolarized resting membrane potential (−61.83±0.41 mV, Fig. 6E) compared to wild-type (−73.46±1.43 mV, Fig. 2D) and is comparable to that of *babo^fd4/df^* and *smox^f4^* animals (−64.44±1.31 mV and −62.67±0.69 mV, respectively). The *daw^1/11^* and *myo^1/4^* mutations had no effect on the resting membrane potential distribution (Fig. 6E). Collectively, these results demonstrate that among Activn/TGF-β ligands, Actβ plays a central role in activating Babo to regulate postsynaptic GluR densities and membrane potential. Since Actβ expression is limited to motor neurons, these results imply that at the NMJ, Actβ signals in an anterograde fashion to regulate GluR densities and membrane potential in contrast to BMP (Gbb) signals that act in a retrograde manner to promote synaptic growth and neurotransmitter release.

### Wit-mediated (BMP) signaling is not important for achieving normal postsynaptic levels of GluRIIA and GluRIIB

**Figure 7:**
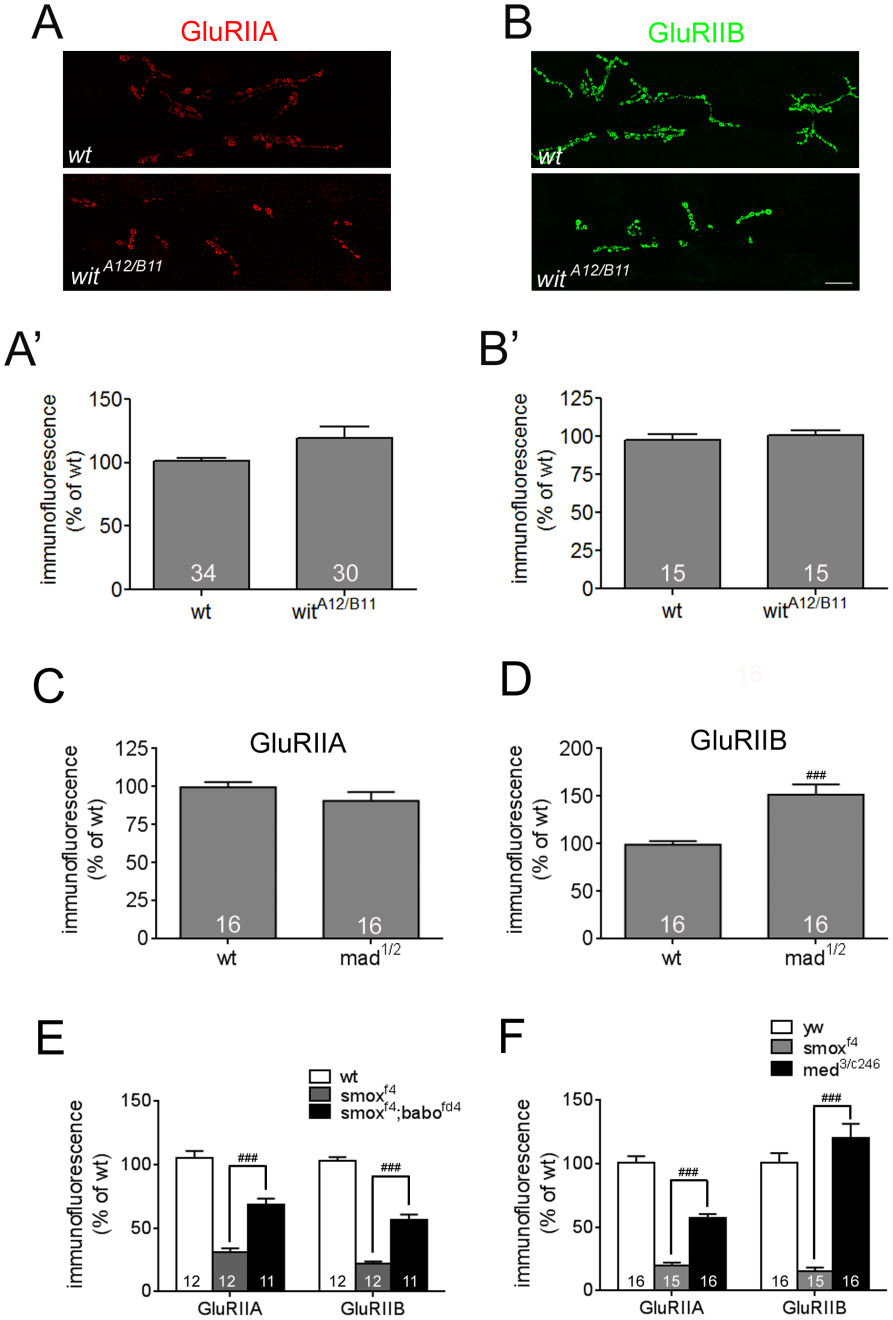
BMP signaling mutants display normal or increased abundance of synaptic GluRs. Representative confocal images of GluRIIA (A) and GluRIIB (B) stainings from wild-type and *wit^A12/B11^* animals. (A′ and B′) Quantification of GluRIIA (A′) and GluRIIB (B′) densities of wild-type and *wit^A12/B11^* animals. (C and D) Quantification of GluRIIA (C) and GluRIIB (D) densities of wild-type and *mad^1/12^* animals. (E) Quantification of synaptic GluRIIA and GluRIIB abundances in *smox* and *smox;babo* double mutants. Introduction of *babo* mutation in the *smox* mutant background increased the GluR levels toward those of the *babo* mutant. (F) Quantification of synaptic GluRIIA and GluRIIB abundances in *smox* and *med* mutants. A mutation in *med* increased the GluR levels from those of *smox* mutant. Error bars represent SEM. ###p<0.001 from unpaired t-test. Scale bar equals 20 µm.

Results from NMJ size assessment demonstrated the differential influences of Activin/TGF-β and BMP pathways on synaptic growth. To determine if the Activin/TGF-β and BMP pathways also have differential influences on the abundances of postsynaptic molecules, we examined the GluRIIA and GluRIIB densities in the BMP pathway mutants, *wit^A12/B11^* and *mad^1/12^*. Consistent with previous results, the *wit^A12/B11^* mutants exhibited a smaller number of synaptic boutons at the NMJ (Fig. 7A and B). The synaptic GluRIIA and GluRIIB densities, however, were not decreased in each bouton of the *witA^12/B11^* animals (Fig. 7A′ and B′). *mad^1/12^* mutants also showed a normal level of GluRIIA at the synaptic boutons (Fig. 7C). Interestingly, the GluRIIB density was increased in *mad^1/12^* mutants (151.6±10.7% of wt, Fig. 7D). These results indicate that BMP signaling is not important or has a slight inhibiting effect in achieving the normal GluR densities. Together with the finding that the Activin/TGF-β pathway positively regulates postsynaptic densities of receptor molecules, these results provide additional evidence that there is a clear segregation in the roles played by the Activin/TGF-β and BMP pathways in synaptic growth and development.

### Loss of Smox protein enhances Mad signaling at the NMJ

We have previously shown that in the wing disc, loss of Smox protein enables Babo to generate an ectopic signal that hyperactivates Mad [50]. As described above, the *smox* loss-of-function phenotype is often signficantly stronger than loss of Babo (Fig. 2, 3 and 4). In additon, the *mad ^1/12^* mutant also exhibits an increase in the GluRIIB density suggesting that Mad maybe acting as a negative regulator of GluR accumulation at the NMJ synapse. This led us to hypothesize that as in the wing disc, loss of Smox in the muscle may also enable Babo to hyperactivate Mad leading to the enhanced loss of GluRs compared to *babo* loss of function alone. To test this hypothesis, we examined the levels of GluRIIA and GluRIIB in *smox;babo* double mutants. We found that simultaneous loss of *babo* and *smox* resulted in a phenotype very similar to loss of *babo* alone (Fig. 7E). In other words loss of *babo* suppressed the stronger *smox* loss of function phenotype just as we observed in the wing disc. As another way to examine if both the Mad and Smox signals participate in regulating GluR densities, we examined the phenotype of *medea* loss. The *medea* locus codes for the sole *Drosophila* co-Smad, therefore its loss should reduce both Smox and Mad signals. Intriguingly, we find that the levels of GluRs were signficantly increased in *medea* mutants compared to *smox* alone again suggesting that Mad and Smox signals regulate GluR levels in a complex manner (Fig. 7F and see below for discussion).

## Discussion

Numerous reports have now implicated the Activin/TGF-β and BMP branches of the TGF-β superfamily in regulating neuronal development, synaptic plasticity and cognitive behavior. Accordingly, members from both subfamilies are widely expressed in the nervous system and are co-expressed in multiple regions of vertebrate and invertebrate brains. It is therefore quite likely that ligands of both subfamilies co-exist within the extracellular space and in some cases, act on the same neurons. Lending support to this idea, pyramidal neurons in the CA3 region of the rat hippocampus are known to accumulate both phosphorylated Smad2 and Smad1/5/8, transcriptional transducers of the canonical Activin/TGF-β and BMP-type signaling, respectively [51], [52]. The activation of these two closely-related signaling pathways in common sets of neurons, or different cells of a common neuronal circuit raises the intriguing question of whether the two pathways play different or redundant roles during neuronal development and function.

In this study, we utilized the *Drosophila* neuromuscular junction to address this issue since ligands of both Activin/TGF-β and BMP families are expressed in both muscle and motor neurons. Our data, together with previous studies on the role of BMP signaling at the NMJ [12]–[16], clearly demonstrate that the two pathways influence NMJ synaptogenesis in different ways. We find that the Activin/TGF-β pathway is necessary for achieving the proper densities of GluRIIA, GluRIIB and Dlg in postsynaptic muscle membrane, while the BMP pathway has a smaller effect on the distribution of these postsynaptic proteins. In addition, the Activin/TGF-β pathway was dispensable for maintaining overall synaptic growth and homeostasis, both of which are strongly affected by mutations in the BMP pathway [12]–[16]. In addition, our tissue-specific rescue experiments indicate that the postsynaptic reception of Activin/TGF-β signaling is important in regulating synaptic GluR abundance, whereas BMP signal reception is known to act in the presynaptic motor neurons to promote synaptic growth [14]. These observations suggest that each pathway influences NMJ synapse development and function by acting mainly in either the pre- or postsynaptic cell.

Interestingly, the BMP and Activin/TGF-β pathways have also been recently found to control different aspects of the *Drosophila* innate immune response [53]. In this case BMP signaling suppresses the expression of multiple antimicrobial peptide genes following wounding, whereas the Activin/TGF-β pathway limits melanization after bacterial infection in adult flies. Therefore, it appears that the division of labor between these subpathways is not limited to just the nervous system, rather it may be the norm when these related signaling pathways act in concert to regulate a common biological process.

The fact that the pathways actually differ in how they affect a complex biological process is not surprising given that the different R-Smads are likely to have different selectivity in gene activation [54]. Within motor neurons, BMP signaling promotes microtubule formation in axons [28] and directly regulates expression of *trio*, a Rac GEF, that acts as a major regulator of actin cytoskeleton in many types of cells [55]. Thus, it is likely that BMP signaling modulates synaptic growth, in part, by changing the structure and dynamics of the actin and microtubule cytoskeleton within motor neurons. BMP signaling also regulates the transcription of *twit*, a gene encoding a L-6 neurotoxin-like molecule that controls the frequency of presynaptic spontaneous vesicle release [56].

Targets of *Drosophila* Activin/TGF–β signaling in any tissue are less well characterized. Within the central brain, glial-derived Myo signals through Smox to control expression of the Ecdysone B1 receptors in remodeling mushroom body neurons [20]. However, it is not clear if EcR-B1 is a direct or indirect target of *smox* transcriptional regulation. It is also unclear if Ecdysone signaling plays a role in regulating synaptogenesis at the NMJ, although it may play a role during metamorphic remodeling of the NMJ as it does for the mushroom body neurons [57]. The only other known targets of Smox are InR, Pi3K and Akt, all of which are Insulin signaling components and are reduced in the *Drosophila* prothoracic gland in the absence of Actvin/TGF-β signaling [27]. Once again the effect may be indirect, but this finding is interesting since Insulin signaling components have been shown to control synaptic clustering of GluRs [58], [59].

### How does Activin/TGF-β influence GluR and Dlg clustering at the NMJ?

The clustering of GluRs and Dlg at the NMJ have been shown to be regulated by both transcriptional and post-transcriptional mechanisms. For example, a recent genetic screen identified *longitudinals lacking (lola)*, a BTN-Zn finger transcription factor, as an essential regulator of GluR and dPak expression in muscles [60]. In contrast, our studies on Activin/TGF-β signaling suggest, at least for GluRIIA, that this pathway functions at the post-transcriptional level since we find that overexpression of *glurIIA-gfp* using an exogenous promotor and transcriptional activator does not lead to an enrichment of GluRIIA^GFP^ at synaptic sites of Activin/TGF-β pathway mutants. This phenotype is reminiscent of that found for certain mutants in the NF-κB signaling system. Loss of Dorsal (an NF-κB homolog), Cactus (an IκB related factor), or Pelle (an IRAK kinase) leads to a substantial reduction of GluRIIA and a slight reduction of Dlg postsynaptic localization at the NMJ and a concomitant reduction in mEJP size [61]. In addition, as we found for loss of Activin/TGF-β signaling, exogenously-expressed GluRIIA-myc did not reach the synaptic surface in NF-κB signaling mutants consistent with a possible role of Activin/TGF-β signaling in regulating NF-κB signaling. However, even if future studies show that the relationship is true, the Activin/TGF-β pathway likely regulates additional factors since its loss also affects GluRIIB levels and muscle resting potential, neither of which is altered in NF-κB pathway mutants. Interestingly, the regulation of GluRIIB levels by Activin/TGF-β signaling does appear to be at the level of transcription, indicating that this signaling pathway likely affects GluR clustering at the NMJ via both transcriptional and post-transcriptional mechanisms.

### Complexity in regulating directional signaling by TGF-β family members at the NMJ

**Figure 8:**
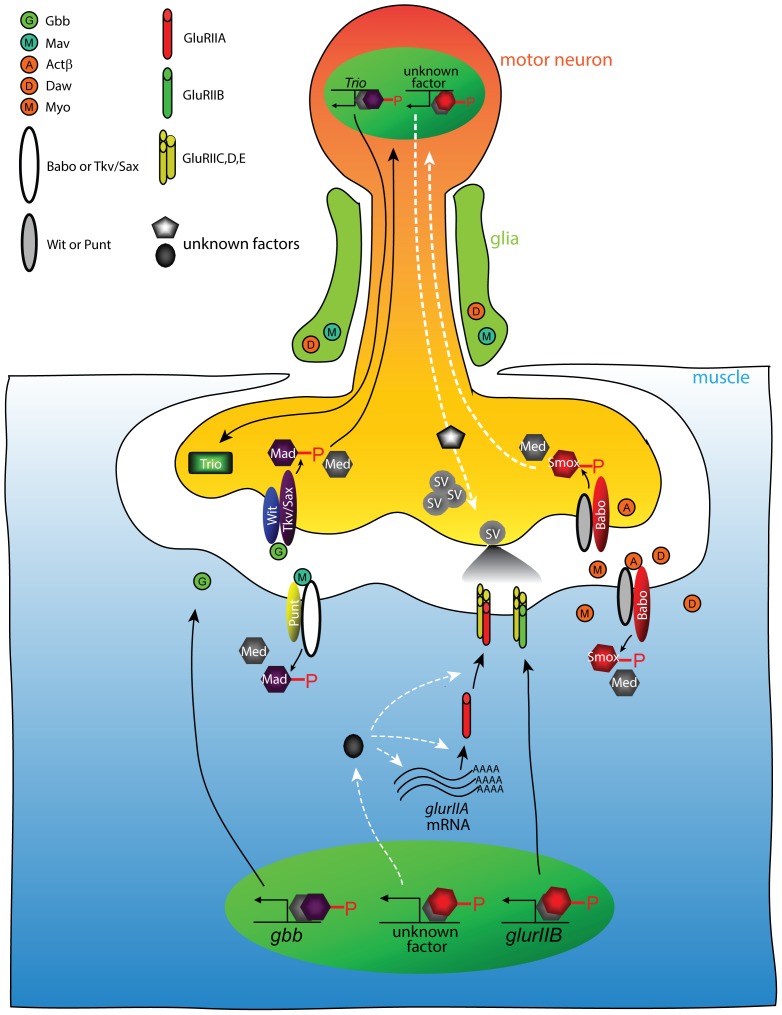
Model of controlling NMJ development and function by Activin/TGF-β and BMP pathways. Activin-type ligands are secreted from glia, motor neuron and muscle. The Activin-type ligands induce Babo-mediated phosphorylation of Smox that facilitates association with Med. In the muscle, the phospho-Smox/Med complexes activate the transcription of *glurIIB* and an unknown factor controlling post-transcriptional process or stability of *glurIIA* mRNA. In the motor neuron, the phospo-Smox/Med complex controls spontaneous release of synaptic vesicles via unknown mechanism(s). On the other hand, glia-secreted Mav stimulates Mad phosphorylation in the muscle resulting in increased *gbb* transcription. Gbb protein is released from the muscle and binds Tkv/Sax and Wit complex on the motor neuron leading to an accumulation of phospho-Mad in the nuclei by an unknown mechanism [68]. The resultant phospho-Mad/Med complex activates the transcription of *trio* whose product promotes synaptic bouton formation. The white dotted arrows in this model depict speculated pathways that should be examined in future studies.

Our analysis of Activin/TGF-β signaling at the NMJ, coupled with previous studies on BMP signaling and the novel ligand Maverick [49], indicates that TGF-β ligands are produced in, and act upon, all three cell types that contribute to NMJ function, specifically the motor neuron, wrapping glia, and muscle (Fig. 8). This leads to the important issue of how directionality of TGF-β signaling at the NMJ is regulated. One possibility is that ligands are sequestered, either inside the secreting cells or on their surfaces, so that they have limited access to receptors on the opposing pre or postsynaptic membrane. For example, Gbb is produced both in muscle and motor neurons [14], leading to the issue of how directional signaling from muscle to motor neurons is achieved. On the postsynaptic muscle, Gbb release is potentiated by dRich, a Cdc42 selective Gap [62] while in the presynaptic neuron Crimpy, a *Drosophila* homolog of the vertebrate Crim1 gene, has been shown to bind to a precursor form of Gbb [63]. The Gbb/Crimpy complex is thought to either interfere with secretion or activation of motor neuron-derived Gbb thus ensuring that only muscle-derived Gbb activates the retrograde BMP signal at the NMJ. Since there are a large number of characterized TGF-β superfamily binding proteins (reviewed in [64]), *Drosophila* homologs of some of these factors such as the BMP binding proteins Cv-2 [65], Sog, Tsg and Dally, or the Activin-binding protein Follistatin, may sequester and regulate levels of active ligands within the NMJ. Sequestering mechanisms may also provide direction control by facilitating autocrine as opposed to juxtacrine signaling. If ligand-binding proteins are associated with the membrane surface of the ligand-producing cell, they may facilitate delivery of the ligand to receptors on the producing cell, thus enhancing autocrine signaling. It is interesting in this regard that in the developing *Drosophila* retina, Actβ appears to signal in an autocrine fashion to control photoreceptor connectivity in the brain [19].

Another important mechanism to control signal direction is likely to be tissue-specific receptor expression. For example, Wit is highly enriched in motor neurons compared to muscle, and this may help ensure that Gbb released from the postsynaptic muscle signals to the presynaptic motor neuron [12], [13]. Type I receptor diversity may be even more important in controlling directionality since at least 2 isoforms of Tkv and three isoforms of Babo have been identified. In the case of Babo, Activin-like ligands have a clear preference for signaling through different receptor isoforms, and these isoforms show differential tissue expression [20], [66].

An additional factor to be considered in understanding TGF-β superfamily signal integration within different NMJ cell types is the possibility of canonical versus non-canonical and/or cross-pathway signaling. For example, in mushroom body neurons Babo can signal in a non-Smad dependent manner through Rho1, Rac and LIM kinase1 (LIMK1) to regulate axon growth and target recognition [67]. Whether this mechanism, or another non-canonical pathway is operative at the NMJ is unclear. Cross-pathway signaling has also recently been identified in *Drosophila*. In this example, loss of Smox protein in the wing disc has been shown to up-regulate Mad activity in a Babo-dependent manner [23], [50]. Double mutants of *babo* and *smox* suppress the cross-pathway signal. As we describe here, *smox* protein null mutations lead to significantly more severe GluR and mEJP defects than strong *babo* mutations alone, and this phenotype is suppressed in double mutants. Thus, as in wing discs, loss of Smox protein likely leads to ectopic Mad activity in muscles that further decrease GluR expression and/or localization at the NMJ. Consistent with this view, we found that loss of Mad actually increases GluRIIB localization, suggesting that Mad acts negatively to regulate GluRIIB in muscle. One possible model to explain the Smox/Mad data is that normally the Babo/Smox signal inhibits Mad signaling which is itself a repressive signal for GluR accumulation. Thus, in *babo* mutants, total GluR levels decrease due to the loss of *smox* and therefore an increase in the repressive Mad signal. In the *smox* protein null mutant even more repressive Mad signal is generated by Babo further hyperactivating Mad activity leading to even lower levels of GluR accumulation. In *medea* mutants the activity of both pathways is reduced thereby returning the level of GluR levels close to normal. Additional experiments employing various single and double mutants, together with tissue-specific expression of various ligands, receptor isoforms and ligand-binding proteins will be needed to fully elucidate how vectorial TGF-β signaling is accomplished at the NMJ. Likewise, the identifcation of directly responding target genes and how they are influencesd by both Smox and Mad signals is needed to fully appreciate how these two TGF-β signaling branches regulates NMJ functional activity.

## Supporting Information

Figure S1 **Normal NMJ size of** ***babo^fd4/df^*** **and** ***smox^f4^*** **mutants assessed by an alternative method that counts the Ib boutons without normalization to muscle surface area.** (A) Dlg staining on muscle 6/7. The fluorescence signal is high at Ib boutons (arrow) and low at Is boutons (arrow head). (A′-A ″) Total Ib bouton numbers at the muscle 6/7 of abdominal segment 2 (A′) and 3 (A″). There was no significant difference in the total Ib number comparing *babo^fd4/df^* or *smox^f4^* to wild-type. Note that the decreased Ib bouton number of *babo^32/52^* is almost (segment 2, A′) and completely (segment 3, A″) restored in *babo^32/df^*animals. (B) Dlg staining on muscle 4 shows higher fluorescence at Ib boutons (arrow) than at Is boutons (arrow head). (B′-B″) Quantification of Ib bouton number at the muscle 4 of segment 2 (B′) and 3 (B″). There was no significant difference in the Ib numbers between the wild-type and *babo^fd4/df^* or *smox^f4^*. Again, heteroallelic combination of *babo^32^* with *babo^df^* rescues the decrease in Ib numbers shown by *babo^32/52^* mutants. Error bars represent SEM. **p<0.01, ***p<0.001 from student t-test. Scale bars equal 20 µm.
(TIFF)

Figure S2 **Defective Activin/TGF-β signaling does not lead to a decrease in activity BMP of the pathway.** (A) p-Mad staining on the cell body of motor neurons in the ventral nerve cords of wild-type and *babo* and *smox* mutants. There was no difference in the intensity of fluorescence. (B) qRT-PCR analysis demonstrates no change in the *gbb* transcript level between wild-type and *babo* or *smox* mutants. Error bars represent SEM. Scale bar equals 40 µm.
(TIFF)

Figure S3 **Quantal content and size relationship and quantal variance.** (A) Plot of quantal content versus quantal size from individual recordings. (B) Quantal variances of wild-type and *babo* and *smox* mutants. The quantal variances were calculated by dividing the standard variation of mEJPs by the mean amplitude in each recording. Error bars represent SEM. ***p<0.001 from One-way ANOVA followed by Dunnett’s multiple comparison test. ###p<0.001 from unpaired t-test.
(TIF)

Figure S4 **Presynaptic development appears to be normal in Activin/TGF-β pathway mutants.** (A) Brp staining on NMJ 6/7 of wild-type and *babo* and *smox* mutants. (A′) Quantification of immunofluorescence of the Brp staining shows no difference. (B) Representative confocal images of Brp staining on NMJ 4. (B′) Normalized number of Brp puncta was not decreased in *babo* and *smox* mutants. Error bars represent SEM. Scale bars equal 20 µm.
(TIF)

Figure S5 **Expression of** ***glurIIA-myc*** **using Mhc enhancer/promotor.** Representative confocal images (A) and quantification (A′) of α-Myc immunoreactivity reveal a lack of GluRIIA^myc^ accumulation at the synapses of *smox* null animals. Error bars represent SEM. ###p<0.001 from unpaired t-test. Scale bars equal 20 µm.
(TIF)

Table S1 **Names and features of ten gene involved in membrane physiology whose transcription profiles were examine in** **Fig. 2** **.**
(TIF)
