# Supplementary figures and images for: Anterograde Activin Signaling Regulates Postsynaptic Membrane Potential and GluRIIA/B Abundance at the Drosophila Neuromuscular Junction
Source: PLoS One. 2014 Sep 25;9(9):e107443. doi: 10.1371/journal.pone.0107443 (PMC4177838; doi:10.1371/journal.pone.0107443)

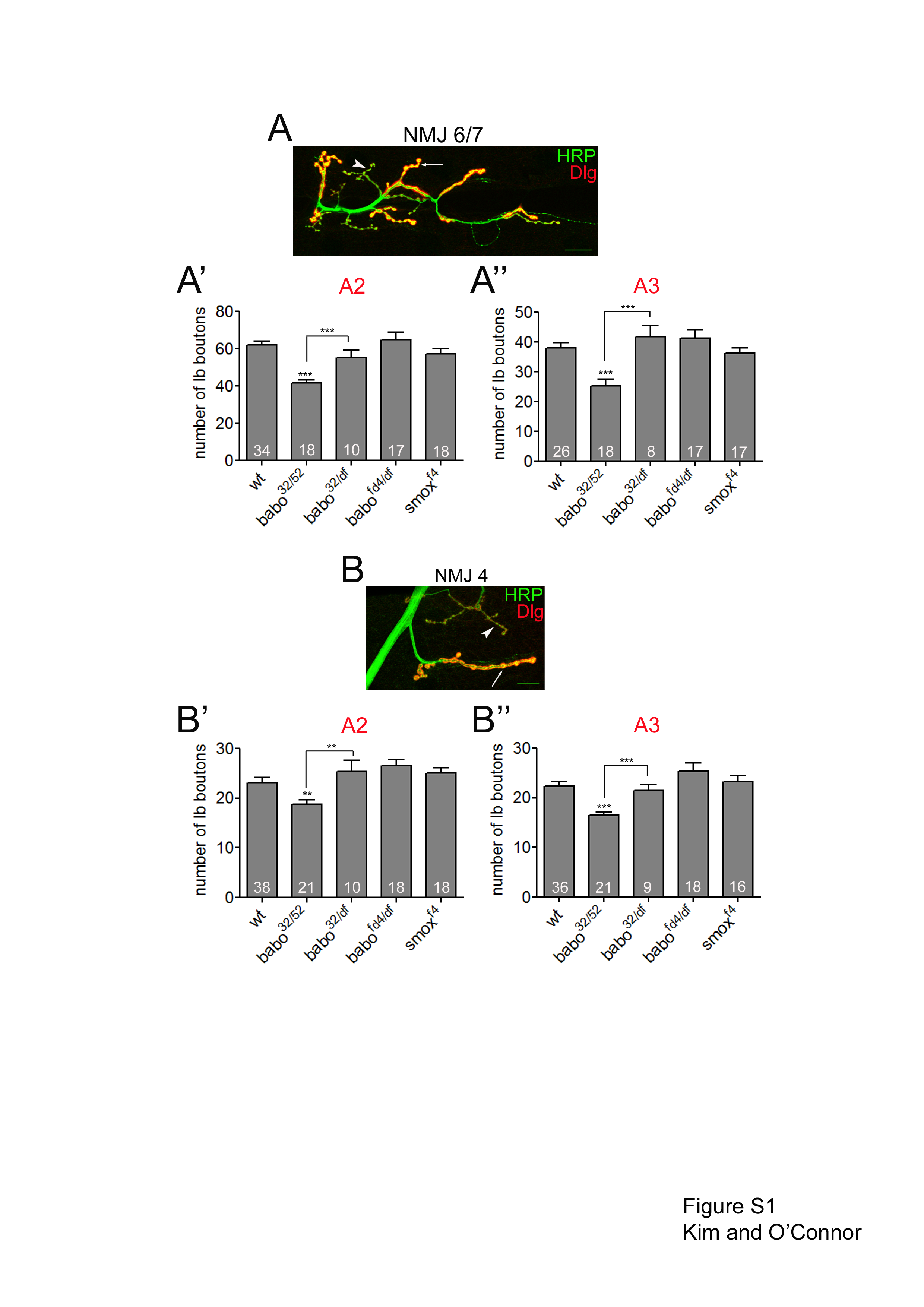

Supplement: Figure S1 — Normal NMJ size of babofd4/df and smoxf4 mutants assessed by an alternative method that counts the Ib boutons without normalization to muscle surface area. (A) Dlg staining on muscle 6/7. The fluorescence signal is high at Ib boutons (arrow) and low at Is boutons (arrow head). (A′-A ″) Total Ib bouton numbers at the muscle 6/7 of abdominal segment 2 (A′) and 3 (A″). There was no significant difference in the total Ib number comparing babofd4/df or smoxf4 to wild-type. Note that the decreased Ib bouton number of babo32/52 is almost (segment 2, A′) and completely (segment 3, A″) restored in babo32/dfanimals. (B) Dlg staining on muscle 4 shows higher fluorescence at Ib boutons (arrow) than at Is boutons (arrow head). (B′-B″) Quantification of Ib bouton number at the muscle 4 of segment 2 (B′) and 3 (B″). There was no significant difference in the Ib numbers between the wild-type and babofd4/df or smoxf4. Again, heteroallelic combination of babo32 with babodf rescues the decrease in Ib numbers shown by babo32/52 mutants. Error bars represent SEM. **p<0.01, ***p<0.001 from student t-test. Scale bars equal 20 µm. (TIFF) [file pone.0107443.s001.tiff]

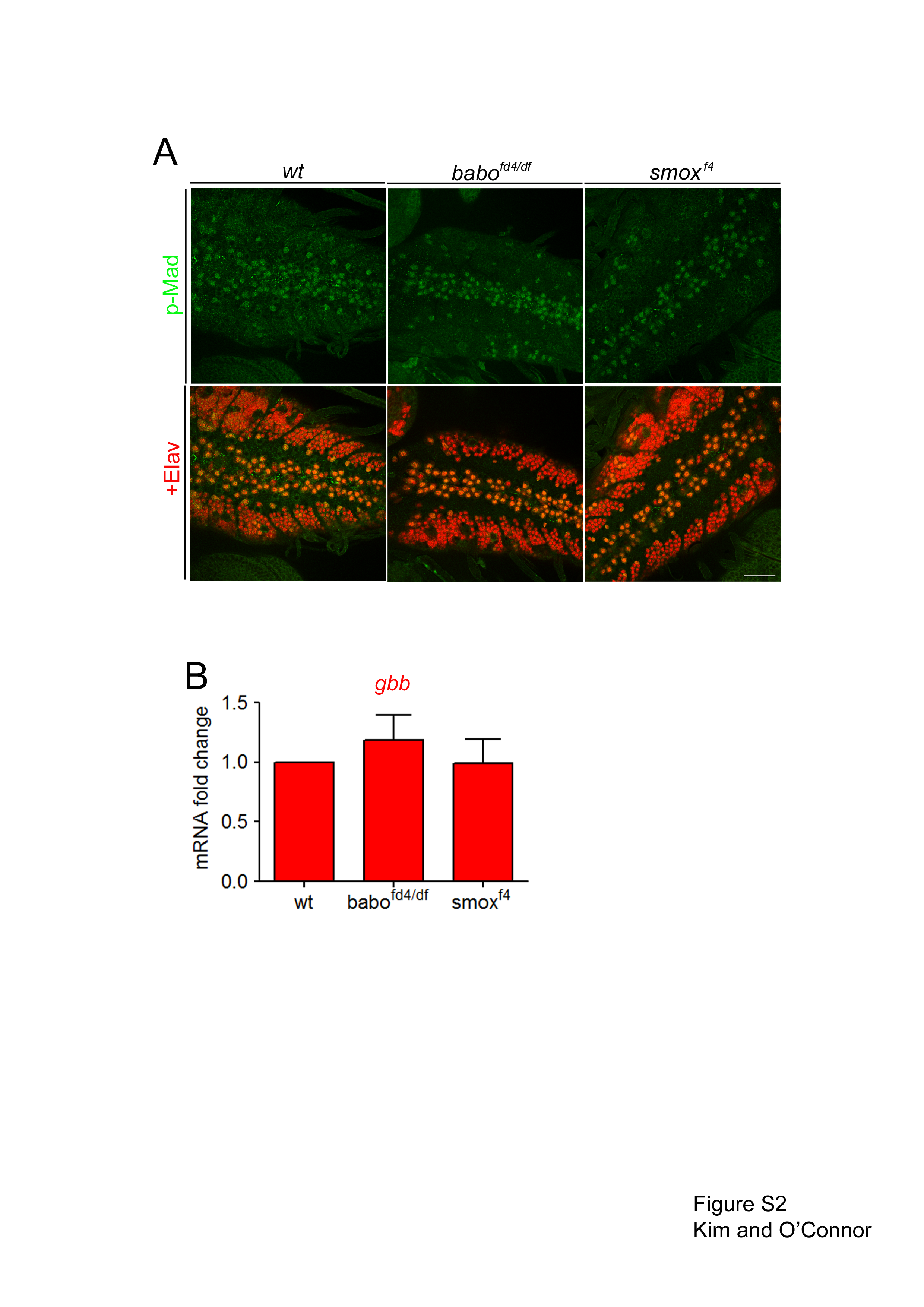

Supplement: Figure S2 — Defective Activin/TGF-β signaling does not lead to a decrease in activity BMP of the pathway. (A) p-Mad staining on the cell body of motor neurons in the ventral nerve cords of wild-type and babo and smox mutants. There was no difference in the intensity of fluorescence. (B) qRT-PCR analysis demonstrates no change in the gbb transcript level between wild-type and babo or smox mutants. Error bars represent SEM. Scale bar equals 40 µm. (TIFF) [file pone.0107443.s002.tiff]

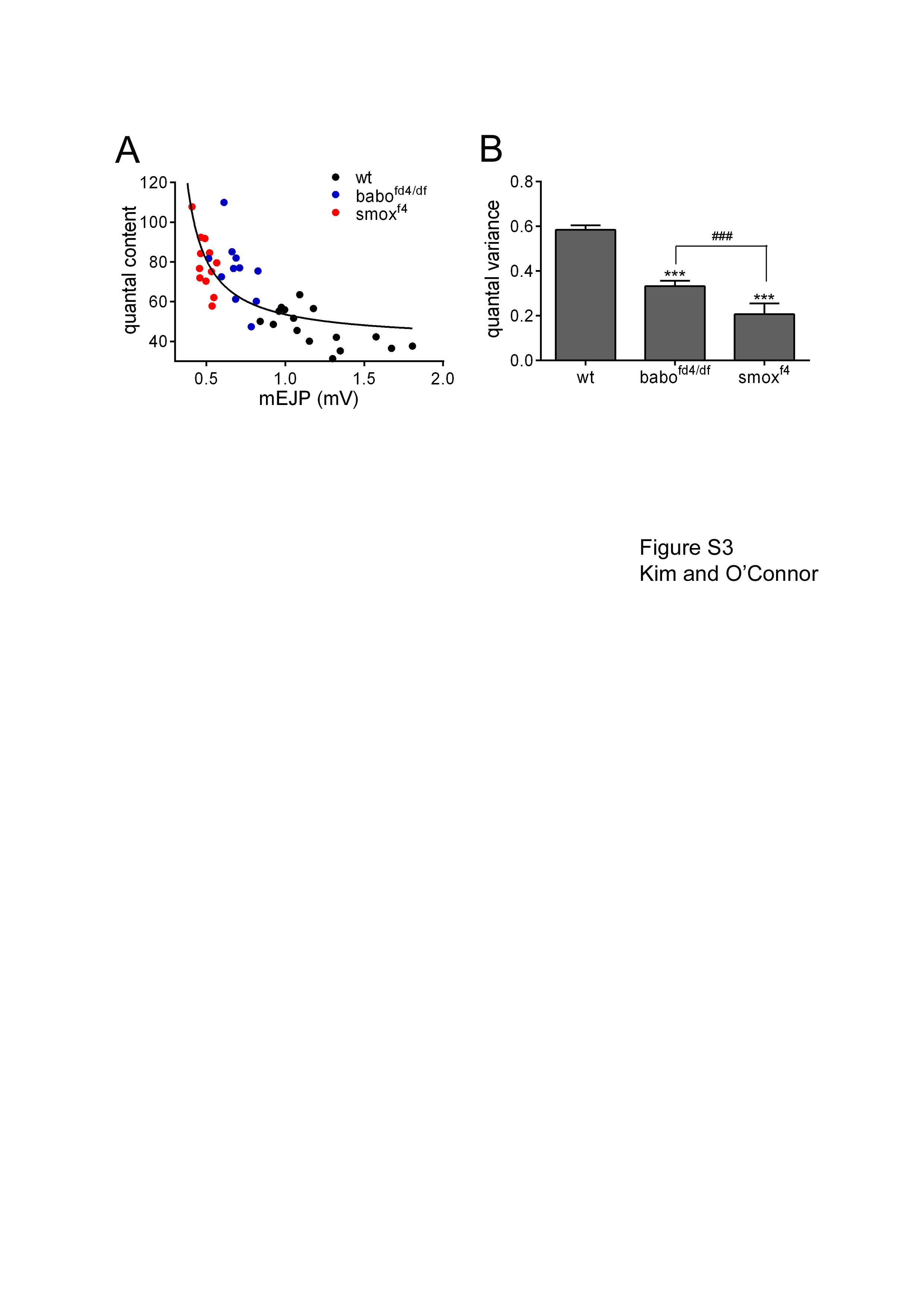

Supplement: Figure S3 — Quantal content and size relationship and quantal variance. (A) Plot of quantal content versus quantal size from individual recordings. (B) Quantal variances of wild-type and babo and smox mutants. The quantal variances were calculated by dividing the standard variation of mEJPs by the mean amplitude in each recording. Error bars represent SEM. ***p<0.001 from One-way ANOVA followed by Dunnett’s multiple comparison test. ###p<0.001 from unpaired t-test. (TIF) [file pone.0107443.s003.tif]

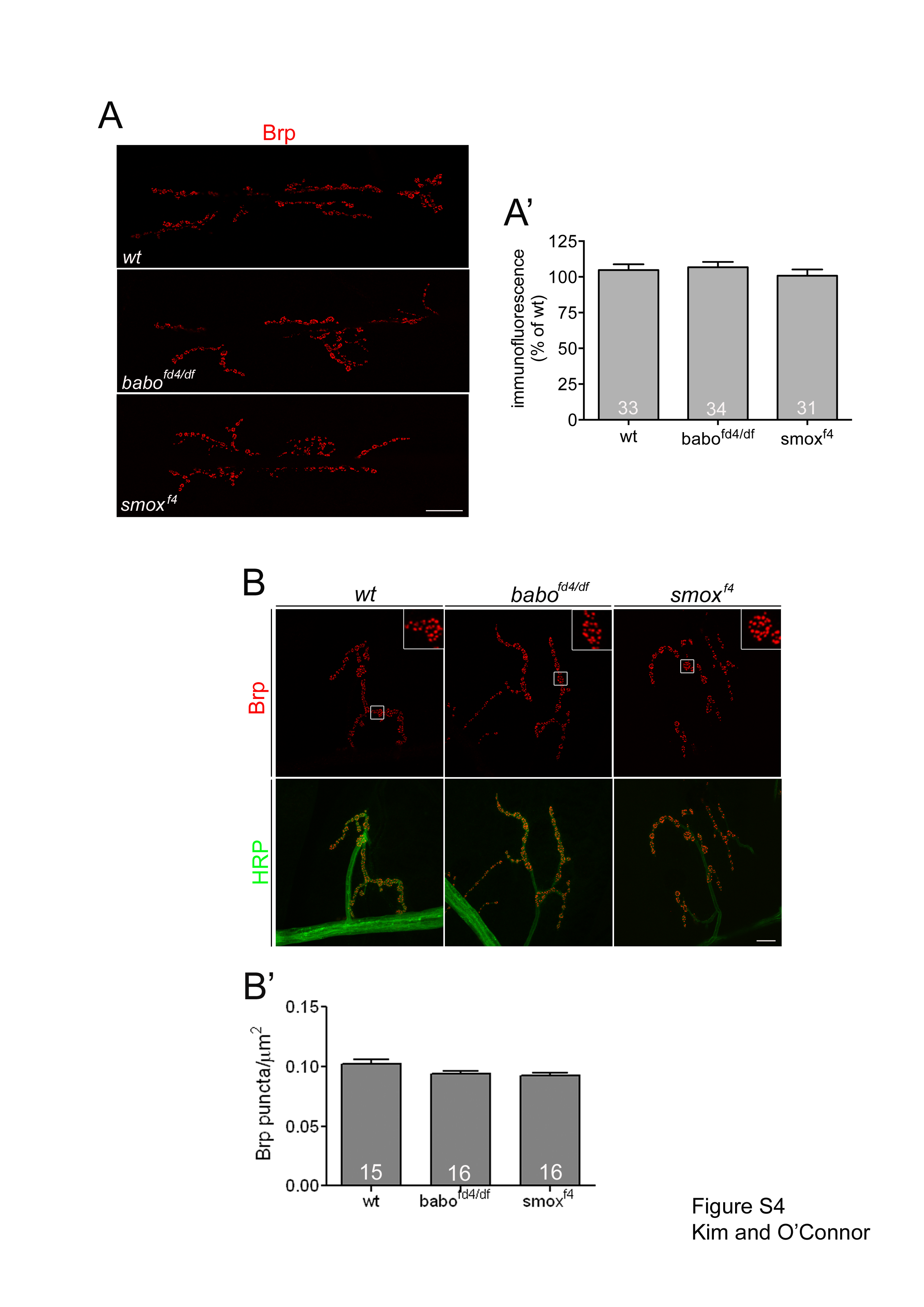

Supplement: Figure S4 — Presynaptic development appears to be normal in Activin/TGF-β pathway mutants. (A) Brp staining on NMJ 6/7 of wild-type and babo and smox mutants. (A′) Quantification of immunofluorescence of the Brp staining shows no difference. (B) Representative confocal images of Brp staining on NMJ 4. (B′) Normalized number of Brp puncta was not decreased in babo and smox mutants. Error bars represent SEM. Scale bars equal 20 µm. (TIF) [file pone.0107443.s004.tif]

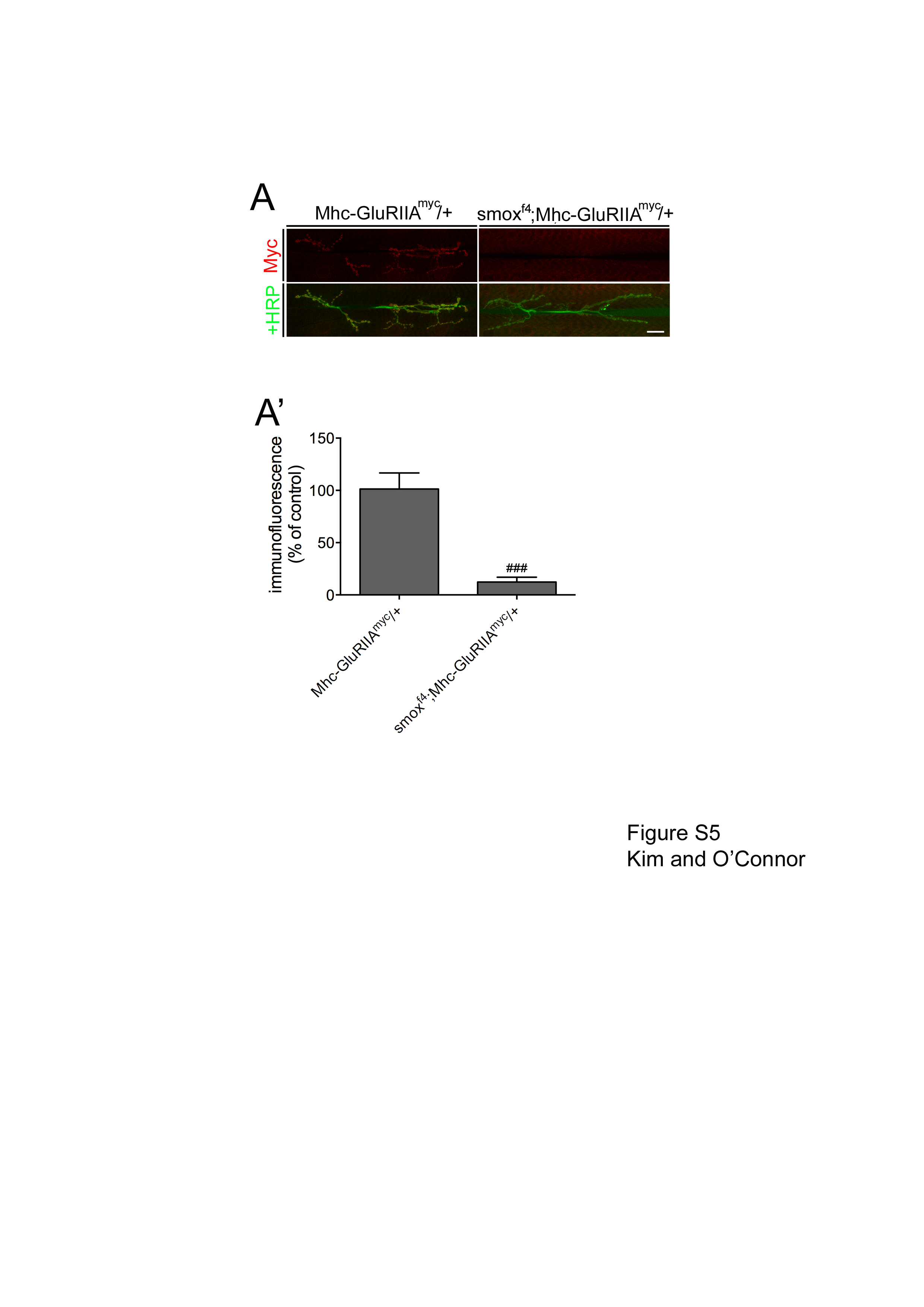

Supplement: Figure S5 — Expression of glurIIA-myc using Mhc enhancer/promotor. Representative confocal images (A) and quantification (A′) of α-Myc immunoreactivity reveal a lack of GluRIIAmyc accumulation at the synapses of smox null animals. Error bars represent SEM. ###p<0.001 from unpaired t-test. Scale bars equal 20 µm. (TIF) [file pone.0107443.s005.tif]

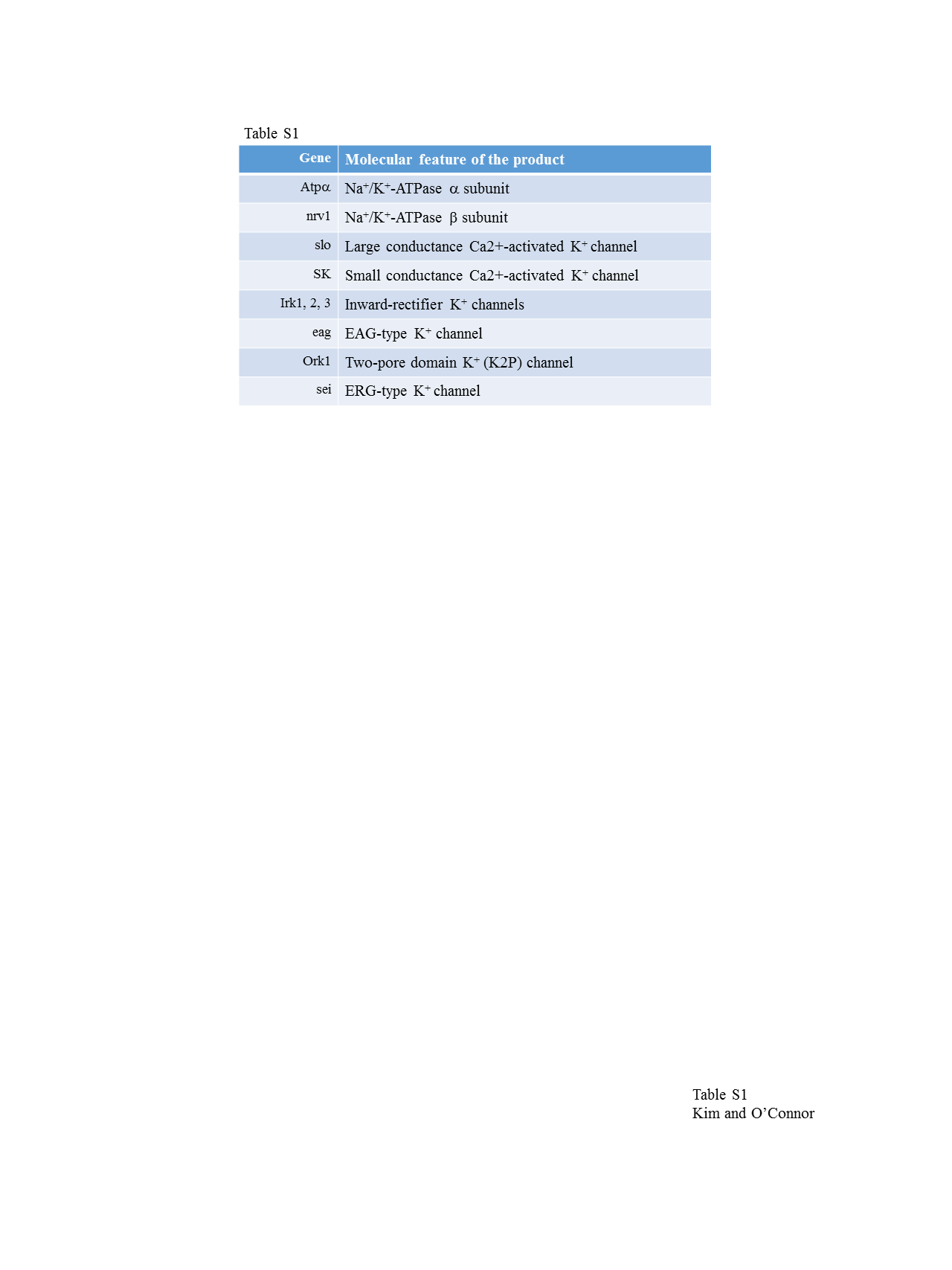

Supplement: Table S1 — Names and features of ten gene involved in membrane physiology whose transcription profiles were examine in Fig. 2 . (TIF) [file pone.0107443.s006.tif]
